# MCM2-7 proteins promote NF-κB transcriptional activity through cooperative promoter recruitment

**DOI:** 10.1042/BSR20260337

**Published:** 2026-06-24

**Authors:** Xian Hong, Ming Sui, Qiang Li, Yan-Ji Lu, Yong-Hong Nie, Yong-Pei Li, Wen-Long Li, Xin-Xin Yue, Jing Xu, Daniel Billadeau, Tao Wang, Zhi-Hui Deng

**Affiliations:** 1 Laboratory of Protein Structure and Function, Institute of Medicine and Pharmacy, Qiqihar Medical University, Qiqihar, Heilongjiang 161006, China; 2 School of Medical Technology, Qiqihar Medical University, Qiqihar, Heilongjiang 161006, China; 3 School of Basic Medicine, Qiqihar Medical University, Qiqihar, Heilongjiang 161006, China; 4 Department of Gastroenterology, The First Hospital of Qiqihar, Qiqihar, Heilongjiang 161006, China; 5 Division of Oncology Research and Schulze Center for Novel Therapeutics, Mayo Clinic College of Medicine, Rochester, MN 55905, U.S.A.; 6 Heilongjiang Provincial key Laboratory of Precise Diagnosis and Neuropsychological Regulation of Mental Disorders, Qiqihar, Heilongjiang 161006, China

**Keywords:** MCM2-7 proteins, NF-κB, RNA polymerase II, transcription initiation

## Abstract

The minichromosome maintenance 2-7 (MCM2-7) proteins form a hexameric protein complex that is the structural core of replicative helicase, which is essential for accurate DNA replication. While their role in replication is well-established, the biological functions of excess chromatin-bound MCM2-7 proteins remain unclear. Here, we identify a transcriptional regulatory function of MCM2-7 through its interaction with nuclear factor κB (NF-κB) p65. We demonstrate that MCM2-7 proteins and NF-κB p65 reciprocally recruit each other to target gene promoters. Consequently, MCM2-7 proteins facilitate the localization of RNA Pol II to NF-κB target gene promoters and along the gene body, thereby promoting transcription of NF-κB target genes. These findings reveal MCM2-7 proteins promote NF-κB target gene transcription by facilitating p65 and RNA Pol II recruitment, offering a potential avenue for fine-tuning NF-κB target gene transcription.

## Introduction

The minichromosome maintenance 2-7 (MCM2-7) proteins are DNA-dependent ATPases, which form hexameric complexes that function as a DNA helicase to unwind DNA duplex templates during DNA replication [1]. The MCM2-7 complex is essential for accurate DNA replication that occurs only once per cell cycle [2]. In the late mitosis and G1 phase, the MCM2-7 hexamer is loaded onto origins of replication with the help of loading factors including ORC, CDC6, and CDT1, forming the inactive head-to-head MCM double hexamers [2]. At the G1-S transition, cyclin-dependent kinase (CDK) and DBF4-dependent CDC7 kinase activities in conjunction with other origin-firing factors convert a fraction of MCM2-7 double hexamers to the active CDC45-MCM-GINS (CMG) helicases that nucleate bidirectional replisome establishment [3]. Moreover, the additional loading of MCM2-7 hexamer onto chromatin is strongly inhibited outside of G1 phase by S-phase CDK activity, which ensures that each origin is fired only once during the cell cycle [4].

Although an excess of MCM2-7 complex is loaded onto chromatin, only about 10% of them give rise to active CMG helicase in the normal S-phase, and the majority of MCM2-7 proteins do not localize to active replication factories in mammalian cells [5]. These observations known as the ‘MCM paradox’ have led to speculation that MCM2-7 proteins may serve other cellular processes. Surplus MCM2-7 complexes remain dormant during normal S-phase progression and are activated to complete replication in response to replicative stress [6]. Therefore, dormant MCM2-7 complexes act as a backup mechanism to maintain genomic stability when cells encounter replication stress. Furthermore, it has been reported that MCM2-7 proteins are involved in gene transcription through association with RNA polymerase II (Pol II) or specific transcription factors such as signal transducer and activator of transcription 1 (STAT1) and hypoxia-inducible factor 1 (HIF-1) [7–10].

The nuclear factor κB (NF-κB) family of transcription factors plays a crucial role in regulating the inducible activation of hundreds of genes that control inflammatory response, cell proliferation and survival [11]. In the classical NF-κB pathway, the NF-κB p65 (also known as RELA) and p50 heterodimer is held inactive in the cytosol by an association with the inhibitor of κB (IκB) [12]. Upon activation by various intracellular and extracellular stimuli such as tumor necrosis factor alpha (TNFα) and lipopolysaccharide (LPS), the IκB kinase complex phosphorylates IκBα leading to its ubiquitylation and proteasomal degradation [12]. The p65-p50 dimer released from IκB inhibition translocates into the nucleus, where it binds to specific cis-regulatory sequences (known as κB sites) in the enhancers or promoters of NF-κB targets and induces the expression of the corresponding mRNA. Our previous study indicates that nuclear FAM21 (WASHC2), a subunit of Wiskott–Aldrich syndrome protein and Scar homologue (WASH) complex that stimulates filamentous actin polymerization and exerts endosomal cargo trafficking [13,14], interacts with p65-p50 dimer and regulates NF-κB transcriptional activity [15]. Furthermore, FAM21 associates with MCM2-7 complex to maintain cell survival under replication stress [16]. Thus, we hypothesize that MCM2-7 proteins might associate with p65-p50 dimer and modulate NF-κB transcriptional activation, which would expand on the biological function of MCM2-7 complex in the transcriptional regulation.

In the present study, we demonstrate that MCM2-7 proteins interact with NF-κB p65 and exhibit mutually dependent co-recruitment to NF-κB target gene promoters. Consequently, MCM2-7 proteins facilitate the localization of RNA Pol II to NF-κB target gene promoters and along the gene body, thereby promoting transcription of NF-κB target genes. Our findings reveal a previously unidentified functional role for MCM2-7 proteins in regulating NF-κB target gene transcription and highlight the interdependent recruitment of MCM2-7 proteins and NF-κB p65.

## Materials and methods

### Reagents and plasmids

The primary antibodies used in the present report were anti-MCM2 (Bethyl Laboratories, A300-191A), anti-MCM3 (Santa Cruz, sc-390480), anti-MCM4 (AssayGenie, CAB13513), anti-MCM5 (Proteintech Group, 11703-1-AP), anti-MCM6 (Thermo Fisher, PA5-35922), anti-MCM7 (Thermo Fisher, PA5-22104), anti-p65 (Santa Cruz, sc-8008), anti-IκBα (Proteintech Group, 66418-1-Ig), anti-p-IκBα (Ser32/36) (Cell Signaling, 9246), anti-RNA Pol II (Millipore, 05-623), anti-RNA Pol II S2p (Diagenode, C15200005), anti-GAPDH (Proteintech Group, 60004-1-lg), anti-α-Tubulin (Thermo Fisher, 62204), anti-BrdU antibody (eBioscience, 11-5071-42), anti-Ku70 (Proteintech Group, 10723-1-AP), anti-HA (Abcam, ab9110), and anti-IgG (Beyotime Biotechnology, A7016). The secondary antibodies were monoclonal mouse Anti-Rabbit IgG-HRP (Jackson ImmunoResearch, 211-032-171), mouse-IgGκ BP-HRP (Santa Cruz, sc-516102), and Alexa Fluor 488-conjugated goat anti-mouse IgG (Thermo Fisher, A-11001). Recombinant human TNFα and Hoechst 33342 were purchased from R&D Systems (210-TA-020/CF) and Thermo Fisher (H1399), respectively. BrdU (5-Bromo-2′-deoxyuridine) and propidium iodide (PI) were purchased from Sigma–Aldrich (B5002 and P4170). Expression plasmid of HA-tagged p65 was constructed by inserting human RELA coding sequence into pCI2.HA vector. The pSpCas9(BB)-2A-Puro (PX459) V2.0 vector (Addgene, 62988) was used to generate gene knockout cell lines, and sgRNAs were inserted as described in previous study [17]. The lentiviral overexpression constructs for full-length MCM4 were generated by seamlessly cloning their complete coding sequences into the pLenti6.3 vector, and for the construction of helicase-deficient mutants, the N-terminal and C-terminal fragments of MCM4 were seamlessly ligated and inserted into the same pLenti6.3 vector backbone via homologous recombination, using the ClonExpress II One Step Cloning Kit (Vazyme, C112-01).

### Cell culture and TNFα stimulation

HeLa, PANC-1, and HEK293T cells were purchased from the Cell Bank of Chinese Academy of Sciences (Shanghai, China). Cells were grown in Dulbecco’s modified Eagle’s medium supplemented with 10% fetal bovine serum in a humidified incubator with 5% CO_2_ at 37°C. For NF-κB activation assays, HeLa cells were treated with TNFα (20 ng/ml) for the indicated period of time.

### RNA Interference and generation of stable cell lines

RNA interference with small interfering RNA (siRNA) was performed using siRNA duplexes obtained from GenePharma. The oligo sequences (listed in the 5′ to 3′ direction) were UUCUCCGAACGUGUCACGUTT (siNC) and GAGCACCAUCAACUAUGAUGAGUUU (sip65). Transfection of siRNA was performed using siRNA-Mate (GenePharma, G04002). The stable MCM2 or MCM6 knockdown cell lines were generated by a lentivirus-mediated expression system as previously described [15]. The shRNA targeting sequences were CATCAGCGACATGTGCAAAGA (shMCM2) and CAGACCATATCCATCACTAAA (shMCM6). CRISPR/Cas9-mediated knockout of MCM2 and MCM4 HeLa cell lines were generated as previously described [18]. The RNA guide sequences were GATCATTGCCTCGCCGACGC targeting exon 2 of the human MCM2 gene and GATGCCAACCTCGCCTGGAG targeting exon 2 of the human MCM4 gene. To validate the genetic alterations induced by CRISPR/Cas9, genomic DNA was extracted from the edited cell populations. Regions flanking the sgRNA target sites were amplified by PCR, and the resulting amplicons were cloned into the pCDNA3.1 vector for single-clone Sanger sequencing. The primers for plasmids construction are listed in the Supplementary Table S1. Stable cell lines expressing full-length MCM4 or a helicase-deleted mutant (MCM4.ΔAAA, lacking the core AAA+ ATPase domain; deletion of residues 458–667) were established in MCM4^KO^ cells using a lentivirus-mediated expression system, essentially as previously described [19].

### Proximity ligation assay

HeLa cells grown on cover slips were fixed, permeabilized, blocked and then subjected to immunofluorescence staining and *in situ* proximity ligation assay (PLA) as previously described [20]. For PLA, the following combination of primary anti-human antibodies were used against: MCM2 (dilution 1:100), MCM6 (dilution 1:100), MCM7 (dilution 1:50), and p65 (dilution 1:20). Hoechst 33342 staining was performed to visualize the nuclei. Fluorescence images were acquired using Zeiss LSM-710 laser scanning confocal microscope integrated with Zen software utilizing 63× oil objective lens and analyzed using ImageJ software.

### Immunoprecipitation, nuclear protein extraction, and western blotting

HEK293T cells were transiently transfected with expression plasmid of HA-tagged p65 or vector. After 48 h transfection, cells with or without TNFα stimulation were harvested and subjected to immunoprecipitation using anti-HA MagBeads (BioMag, BMHA-1). Nuclear and cytosolic fractionated protein extracts were prepared as previously described [18]. Meanwhile, 40–50 μg of protein extracts were prepared and analyzed by western blotting. The protein signals were detected with Amersham Imager 680 (GE Healthcare Life Sciences).

### RNA-Seq analysis

Wild type (WT) and MCM4^KO^ cells were treated with TNFα (20 ng/ml) for 1 h. RNA extraction and RNA-seq analysis were performed by Sangon Biotech. RNA from NC, NCT, M4 and M4T groups was isolated using TRIzol reagent. Sequencing was performed on a MGISEQ-T7 platform. The differentially expressed genes (DEGs) were filtered based on *P-*values <0.05 and fold-changes ≥2.0. The Minus-average (MA) plot, Gene ontology enrichment, Volcano plot, and Gene set enrichment analysis (GSEA) were plotted by https://www.bioinformatics.com.cn for data visualization.

### RNA extraction and quantitative real-time PCR

Total RNA was isolated using the RNAprep Pure Cell/Bacteria Kit (TIANGEN, DP430), and cDNA was generated using the FastKing Reverse Transcription Kits (TIANGEN, KR118). Relative mRNA levels, normalized to GAPDH, were determined using SGExcel FastSYBR Master Mixture (Sangon Biotech, B532955) in an Applied Biosystems QuantStudio 3 Real-Time PCR Systems. Primer sequences are presented in Supplementary Table S2.

### Chromatin immunoprecipitation

Chromatin immunoprecipitation (ChIP) was performed using the EZ-Magna ChIP kit (Millipore, 17-10086) according to the manufacturer’s instructions. Isolated chromatin was sonicated by Bioruptor Pico (Diagenode) to create a smear of DNA in the range of 200–1000 bp. The primer sequences used for ChIP quantitative real-time PCR (qRT-PCR) are provided in Supplementary Table S3.

### Cell cycle analysis by BrdU incorporation

BrdU incorporation was performed as previously described [21]. Briefly, the cells grown in the 6-well plates were treated with 30 μM BrdU for 2 hr, and then were fixed, acid-treated, and detected using FITC-conjugated anti-BrdU antibody (eBioscience, 11-5071-42). The DNA was stained with 20 μg/ml of PI. All samples were analyzed using a FACS Calibur flow cytometer (BD Biosciences) and FlowJo software.

### Statistical analysis

Statistical analysis of all data was conducted using GraphPad Prism 8.0. Two-tailed Student’s *t-*tests were used for statistical analysis between two groups, and one-way analysis of variance (ANOVA) with Dunnett’s multiple comparisons test was used for multiple groups. Data were reported as mean ± the standard deviation (SD), with *P*<0.05 considered statistically significant. *P<0.05, **P<0.01, ***P<0.001, ****P<0.0001; ns, not significant.

## Results

### MCM2-7 proteins interact with NF-κB p65

**Figure 1:**
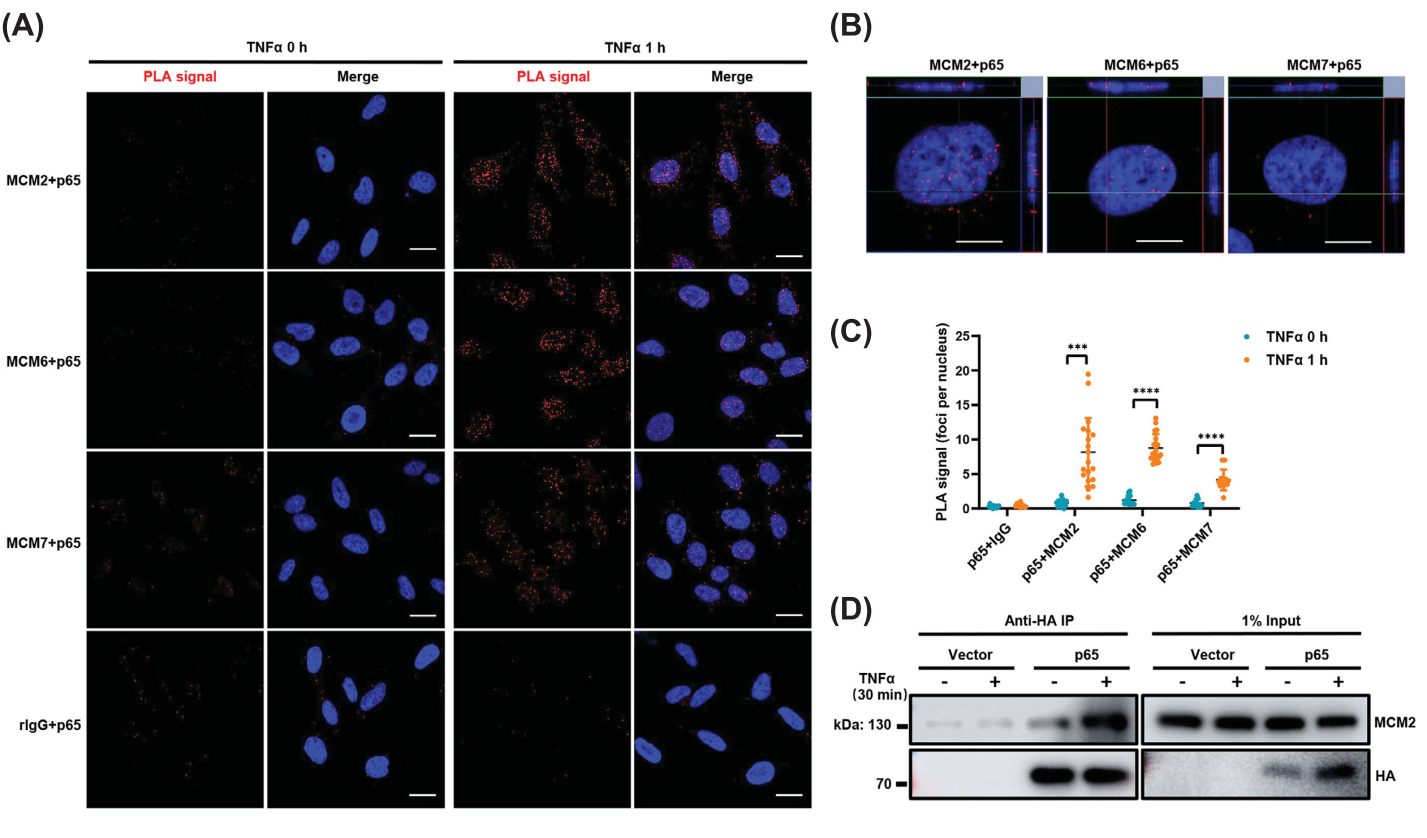
MCM2-7 proteins interact with NF-κB p65 (**A**) HeLa cells with 20 ng/ml TNFα treatment for 1 h or not were subjected to PLA using the indicated primary antibodies. Scale bars: 20 μm. (**B**) Confocal Z-stack images and orthogonal views were acquired for each group of treated cells. Scale bars: 10 μm. (**C**) PLA signal foci numbers per nucleus for 105–168 cells of each group were calculated and presented as mean ± SD. Unpaired *t-*test was performed between TNFα-treated and untreated groups. ***P<0.001; ****P<0.0001. (**D**) HA-tagged p65 was expressed in HEK293T cells and subsequently immunoprecipitated from cell lysate with HA antibodies. *n* = 3 biological replicates.

To determine whether in fact the MCM2-7 complex interacts with NF-κB proteins, we performed an *in situ* PLA by co-staining MCM proteins such as MCM2, MCM6, or MCM7 and p65 NF-κB subunit (Figure 1A–C). The specificity of the anti-MCM2 and anti-MCM6 antibodies used in these assays was confirmed by immunofluorescence staining in cells depleted of MCM2 or MCM6, which showed a marked reduction in signal relative to control cells (Supplementary Figure S1). Although minimal PLA signals were detected in unstimulated cells, robust nuclear PLA signals (red signals) were observed in HeLa cells after TNFα treatment (Figure 1A–C), indicating TNFα-induced MCM2-7/NF-κB interaction. This interaction was further confirmed by co-immunoprecipitation assays, where ectopically expressed HA epitope tagged p65 co-immunoprecipitated endogenous MCM2 from HEK 293T cells, with enhanced interaction upon TNFα stimulation (Figure 1D). These results suggest that the interaction of MCM2-7 components with NF-κB is inducibly enhanced upon TNFα stimulation.

### MCM4 promotes NF-κB target gene transcription

**Figure 2:**
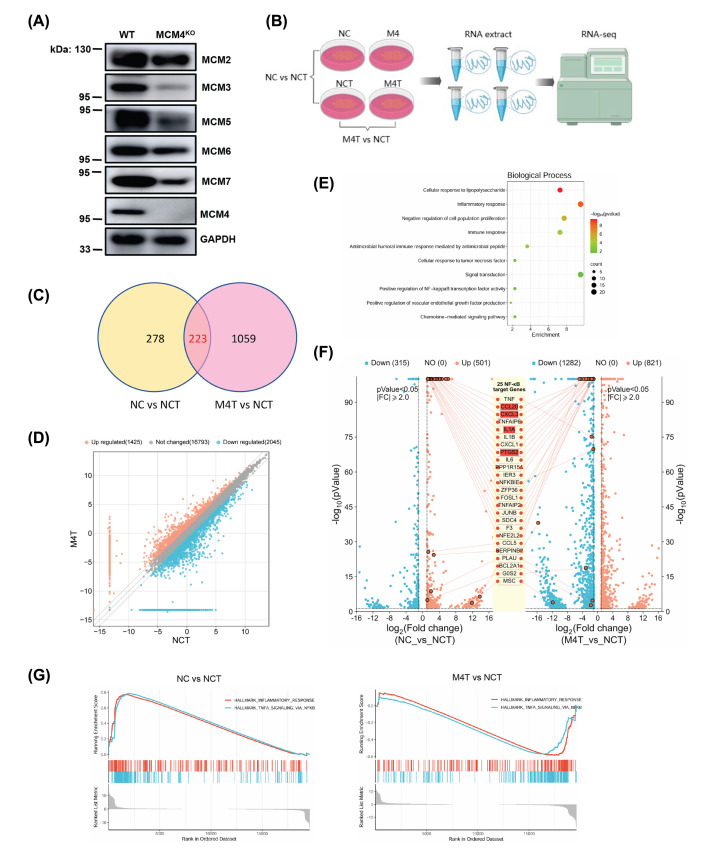
MCM4^KO^ attenuates the transcription of NF-κB target genes (**A**) Expression of the indicated MCM2-7 subunits were examined by western blotting in HeLa MCM4^KO^ cell lines. (**B**) Experimental workflow for RNA sequencing analysis. (**C**) Venn diagram indicating the intersection between DEGs in TNFα-treated versus untreated WT cells (NCT versus NC) and TNFα-treated MCM4^KO^ versus TNFα-treated WT cells (M4T versus NCT). (**D**) MA plot showing global changes in gene expression following MCM4 depletion. (**E**) Gene Ontology biological process classification of the 223 coregulated genes. (**F**) Volcano plot displaying genes detected by RNA-seq, the 25 NF-κB target genes within the intersecting cluster of DEGs are shown. (**G**) GSEA analysis of RNA-seq data. NC: WT cells without TNFα treatment; NCT: WT cells with 20 ng/ml TNFα treatment for 1 h; M4: MCM4^KO^ cells without TNFα treatment; M4T: MCM4^KO^ cells with 20 ng/ml TNFα treatment for 1 h.

The observed interaction between MCM2-7 proteins and NF-κB p65 prompted us to investigate the role of MCM2-7 proteins in NF-κB transcriptional activity. To test this, we generated MCM4 knockout (MCM4^KO^) cells. MCM4 was selected because its central location between MCM6 and MCM7 within the core catalytic module renders it critical for the structural integrity of the MCM2-7 hexamer. Sanger sequencing and qRT-PCR analyses confirmed the introduction of frameshift mutations and subsequent nonsense-mediated mRNA decay, leading to the complete depletion of MCM4 (Supplementary Figure S2A,B). Notably, the loss of MCM4 concurrently resulted in a marked reduction in the steady-state levels of other MCM2-7 subunits, aligning with the established interdependence of these proteins for hexameric stability (Figure 2A). To evaluate transcriptomic alterations, WT and MCM4^KO^ cells were stimulated with or without TNFα and subjected to RNA-seq (Figure 2B). A total of 501 genes were found to be up-regulated upon TNFα treatment in WT cells (Figure 2C). Strikingly, 223 of these TNFα-responsive genes showed significantly reduced expression in MCM4^KO^ cells compared with WT cells (Figure 2C). An MA plot confirmed that global transcription remained largely unperturbed following MCM4 depletion, with the vast majority of genes showing no significant changes in expression (Figure 2D). Gene ontology analysis demonstrated enrichment of these 223 genes in inflammatory and immune response pathways, which are known to be regulated by NF-κB (Figure 2E). Notably, these 223 genes included 25 well-established NF-κB target genes (Figure 2F). GSEA confirmed MCM4^KO^ led to reduced expression of most of genes involved in inflammatory response and TNFα signaling via NF-κB, which were enhanced by TNFα treatment in WT cells (Figure 2G). Collectively, these results indicated MCM4 is required for optimal transcription of NF-κB target genes.

### MCM2-7 proteins promote the expression of NF-κB target genes

**Figure 3:**
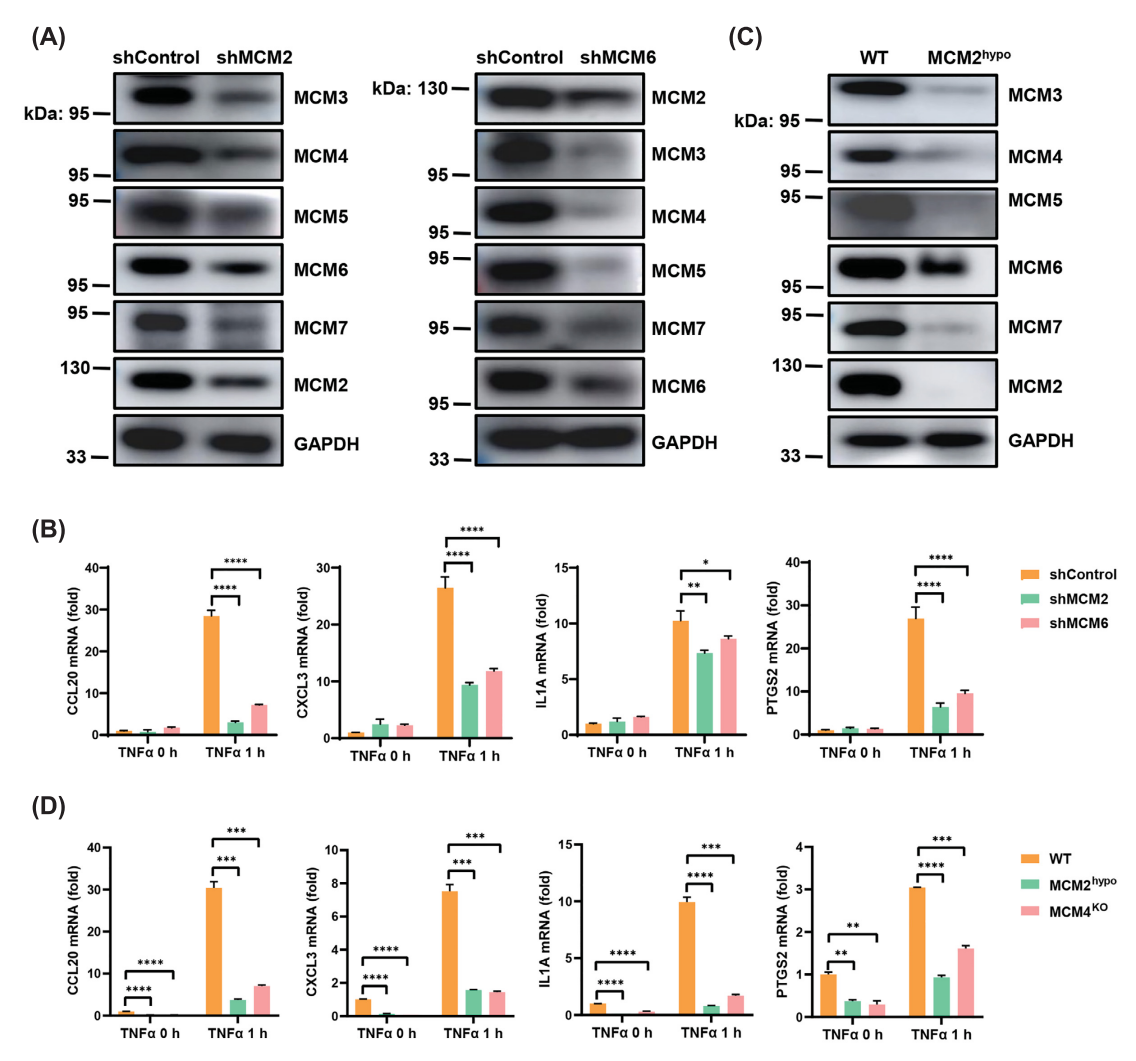
Loss of MCM2-7 proteins reduces the expression of NF-κB target genes (**A**) Expression of the indicated MCM2-7 subunits were examined by western blotting in HeLa stable cells. (**B**) qRT-PCR was performed to analyze the basal and TNFα-induced mRNA expression of selected NF-κB targets. Data are presented as mean ± SD. *P<0.05; **P<0.01; ****P<0.0001. One-way ANOVA with Dunnett’s multiple comparisons test was performed among shControl, shMCM2 and shMCM6. (**C**) Expression of the indicated MCM2-7 subunits were examined by western blotting in HeLa MCM2^hypo^ cell line. (**D**) The expression of NF-κB target genes was analyzed by qRT-PCR in HeLa MCM2^hypo^ and MCM4^KO^ cells in the presence or absence of TNFα stimulation. Data are presented as mean ± SD. **P<0.01; ***P<0.001; ****P<0.0001. One-way ANOVA with Dunnett’s multiple comparisons test was performed among WT, MCM2^hypo^ and MCM4^KO^. *n* = 3 biological replicates.

To further confirm whether MCM2-7 complex affects NF-κB transcriptional activity, we generated stable MCM2 or MCM6 knockdown HeLa cell lines using lentiviral shRNA constructs (Figure 3A). Depletion of either subunit reduced the protein levels of all examined MCM2-7 components, underscoring the mutual interdependence of the hexameric complex. As shown in Figure 3B, knockdown of MCM2 or MCM6 significantly reduces the TNFα-induced expression of the mRNAs for several NF-κB target genes, including those encoding CCL20, CXCL3, IL1A, and PTGS2. To rigorously investigate the function of the MCM2-7 complex, we employed CRISPR/Cas9 to disrupt the MCM2 locus. Genotyping and transcript analysis revealed a compound heterozygous mutant (Supplementary Figure S2C,D): one allele harbors a frameshift insertion, while the other carries a 42-bp in-frame deletion that removes the anti-MCM2 antibody epitope (residues 14–28). Consequently, although immunoblotting failed to detect MCM2 protein (Figure 3C), a truncated protein was presumably expressed from the deletion allele. Thus, we designated this cell line as a hypomorphic MCM2 mutant (MCM2^hypo^). Notably, despite retaining partial MCM2 function, the steady-state levels of other MCM2-7 subunits were markedly reduced in MCM2^hypo^ cells (Figure 3C), indicating that even partial disruption of the complex compromises its overall stability. Consistently, both MCM2^hypo^ and MCM4^KO^ cells displayed a marked decrease in basal and TNFα-dependent expression of NF-κB targets compared to WT cells (Figure 3D). Notably, BrdU incorporation assay demonstrated that depletion of MCM2, MCM6, or MCM4 did not detectably alter normal cell cycle progression (Supplementary Figure S3). This suggests that the observed effects of MCM2-7 proteins on NF-κB gene transcription are unlikely to arise from disrupted DNA replication.

To verify that the transcriptional defects observed in MCM4^KO^ cells are specifically attributable to MCM4 loss, we performed rescue assays. WT MCM4 cDNA was stably reintroduced into MCM4^KO^ cells via lentiviral transduction. In parallel, to assess whether the helicase-associated domain contributes to the transcriptional function of MCM4, we generated a mutant lacking the core AAA+ ATPase domain (MCM4.ΔAAA: deletion of residues 458–667) and stably expressed it in MCM4^KO^ cells (Supplementary Figure S4A). Reconstitution with full-length MCM4 partially restored the TNFα-induced expression of NF-κB target genes (Supplementary Figure S4B). Although the ΔAAA mutant also conferred partial rescue, its efficiency was markedly lower than that of the full-length protein, indicating that the AAA+ domain is required for the optimal function of MCM4 in this transcriptional context.

NF-κB is well established to be constitutively activated in many types of cancer [11], and more than 80% of human pancreatic cancer cell lines showed constitutively activated p65 [22]. We thus tested whether MCM2-7 complex affects NF-κB target gene transcription in pancreatic cancer cells. Depletion of MCM2 in PANC-1 pancreatic cancer cells diminished the basal mRNA levels of several NF-κB targets such as CCL2, CCL20, CXCL3, EFNA1, and IL1A (Supplementary Figure S5A,B). These data indicate that MCM2-7 proteins are crucial for NF-κB dependent gene expression.

### MCM2-7 proteins do not affect p65 nuclear translocation and expression

**Figure 4:**
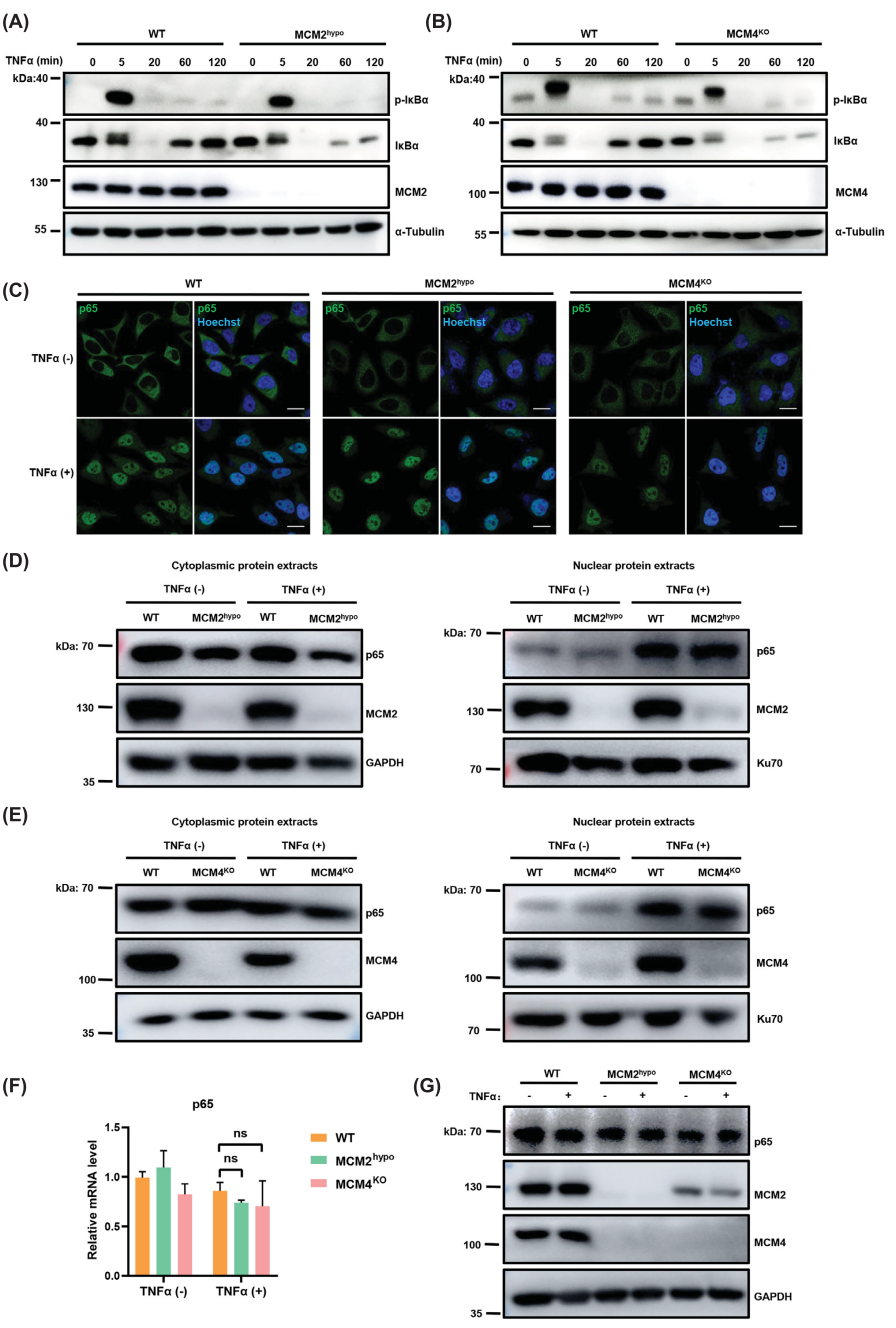
MCM2-7 proteins do not affect p65 nuclear translocation and expression MCM2^hypo^ (**A**) or MCM4^KO^ (**B**) and WT HeLa cells were treated with TNFα for the indicated periods of time, and then the phosphorylated (p-IκBα) and total IκBα levels were analyzed by western blotting. (**C**) HeLa cells were treated with TNFα for 30 min, and endogenous p65 distribution was determined by immunofluorescence. Scale bars: 20 μm. Cytosolic and nuclear protein extracts of MCM2^hypo^ (**D**) and MCM4^KO^ (**E**) as well as WT HeLa cells were analyzed by western blotting. GAPDH and Ku70 were used as cytoplasmic and nuclear markers, respectively. (**F**) The mRNA expression of p65 was measured in WT, MCM2^hypo^, and MCM4^KO^ HeLa cells by qRT-PCR. Data are presented as mean ± SD. One-way ANOVA with Dunnett’s multiple comparisons test was performed among WT, MCM2^hypo^, and MCM4^KO^. ns, not significant. (**G**) The protein expression of p65 was tested by western blotting.

A key step in canonical NF-κB activation is nuclear translocation of p65-p50 heterodimer following the degradation of IκB proteins. To address how MCM2-7 proteins affect NF-κB transcriptional activity, we first assessed the kinetics of phosphorylation and degradation of IκBα in knockout cells. Exposure to TNFα resulted in a substantial IκBα phosphorylation by 5 min and loss of total IκBα protein by 20 min. Loss of MCM2 or MCM4 had no effect on either the kinetics of phosphorylation of IκBα or its subsequent degradation (Figure 4A,B). However, at 60 and 120 min post-stimulation, IκBα protein levels remained aberrantly low in both MCM2^hypo^ and MCM4^KO^ cells compared to WT controls. Transcript analysis revealed that TNFα-induced NFKBIA (IκBα) mRNA expression was significantly blunted in MCM-deficient cells at 60 min (Supplementary Figure S6). Since IκBα is a well-established transcriptional target of NF-κB, this impaired mRNA induction accounts for the diminished IκBα protein resynthesis observed at later time points. It also provides additional evidence that MCM2-7 function is required for optimal NF-κB transcriptional activity.

We then assessed whether p65 would translocate from the cytosol to the nucleus in the absence of MCM2-7 complex. Immunofluorescence showed that in unstimulated cells p65 located in the cytosol and moved to the nucleus upon TNFα stimulation (Figure 4C). MCM2^hypo^ and MCM4^KO^ cells displayed the same extent of p65 translocation upon TNFα exposure compared with WT cells, and no differences were observed. Consistently, the subcellular fractionation data indicated that MCM2 or MCM4 deficiency did not interfere with p65 nuclear translocation (Figure 4D,E). In addition, loss of MCM2 or MCM4 did not change the mRNA and protein levels of p65 (Figure 4F,G). These results suggest that MCM2-7 proteins regulate NF-κB activity downstream of p65-p50 nuclear translocation.

### MCM2-7 proteins and NF-κB p65 reciprocally recruit each other to target gene promoters

**Figure 5:**
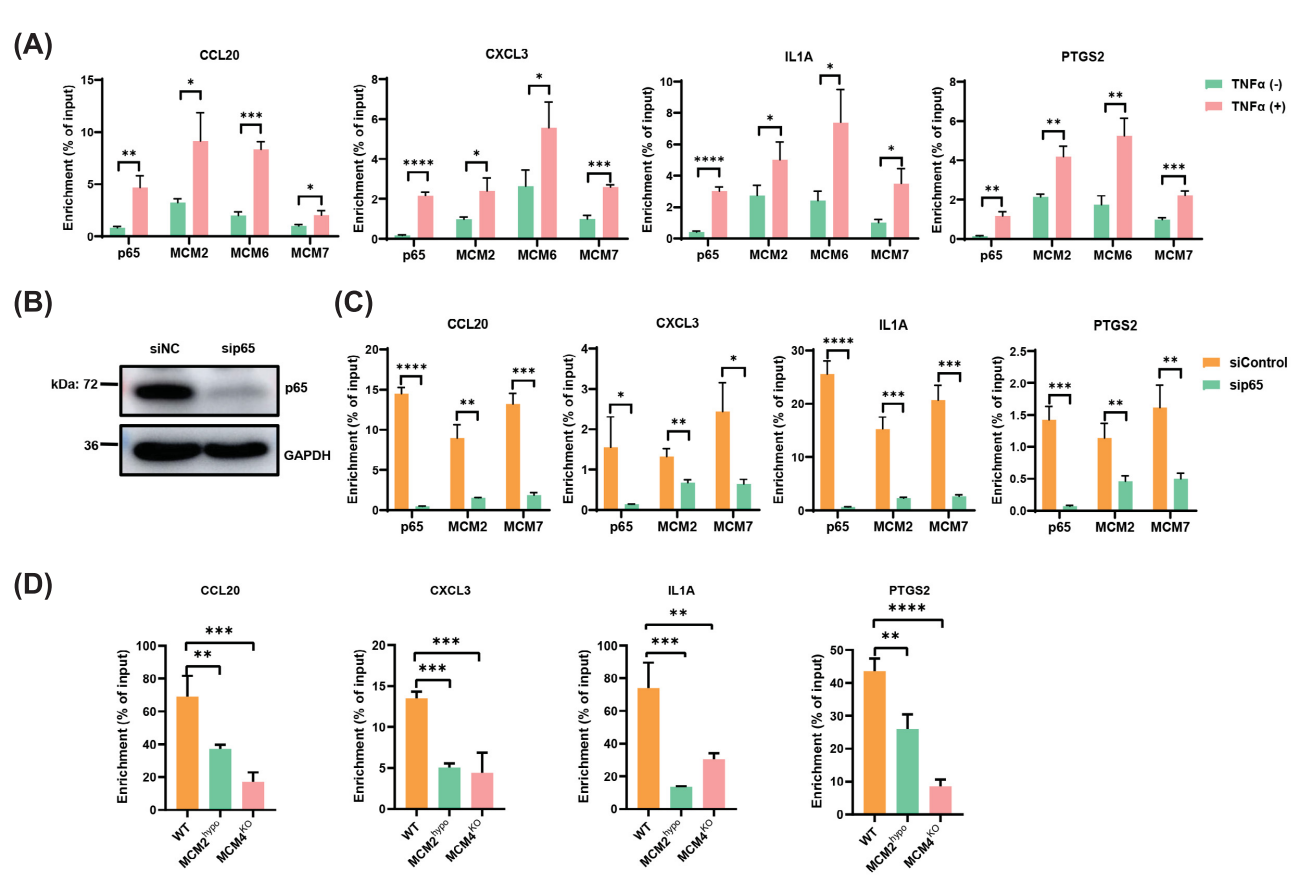
MCM2-7 complex and p65 mutually recruit each other to NF-κB target gene promoters (**A**) HeLa cells were treated with or without TNFα for 30 min, and the promoters of selected NF-κB targets were analyzed by ChIP assays with the indicated antibodies. Data are presented as mean ± SD. Unpaired *t*-test was performed between treated and untreated groups. *P<0.05; **P<0.01; ***P<0.001; ****P<0.0001. (**B**) Expression level of p65 in HeLa cells transfected with either control siRNA (siNC) or siRNAs targeting p65 (sip65) was examined by western blotting. (**C**) HeLa cells transfected with either siNC or sip65 were treated with TNFα for 30 min, and then ChIP assays were performed to examine the promoters of NF-κB targets with the indicated antibodies. Data are presented as mean ± SD. Unpaired *t-*test was performed between siNC and sip65 groups. *P<0.05; **P<0.01; ***P<0.001; ****P<0.0001. (**D**) MCM2^hypo^ and MCM4^KO^ as well as WT HeLa cells were treated with TNFα for 30 min followed by ChIP assays with anti-p65 antibody. Data are presented as mean ± SD. One-way ANOVA with Dunnett’s multiple comparisons test was performed among WT, MCM2^hypo^ and MCM4^KO^. **P< 0.01; ***P< 0.001; ****P<0.0001. *n* = 3 biological replicates.

Previous studies have demonstrated that MCM2-7 proteins can be recruited to STAT1 target gene promoters in an inducible manner [9]. To determine whether the MCM2-7 complex is involved in NF-κB mediated transcription activation, the MCM2-7 recruitment at the promoter of NF-κB target genes was examined by ChIP assays. As expected, TNFα stimulation produced a strong increase in p65 recruitment to the κB site-containing promoters of NF-κB targets such as CCL20, CXCL3, IL-1A, and PTGS2 (Figure 5A). Interestingly, in resting cells, a fraction of MCM proteins, including MCM2, MCM6, and MCM7, bound to these NF-κB binding sites, which is consistent with a previous report that MCM2-7 complex is enriched at active gene promoters [23]. Moreover, the recruitment of these MCM proteins to the promoters was dramatically enhanced after TNFα treatment (Figure 5A). Importantly, depletion of p65 by siRNA markedly decreased the loading of MCM2 and MCM7 onto the NF-κB target gene promoters in the presence of TNFα stimulation (Figure 5B,C), suggesting that NF-κB is required for inducible recruitment of MCM2-7 complex to κB sites. Conversely and notably, depletion of either MCM2 or MCM4 reduced the binding of p65 to the promoters of NF-κB target genes upon induction (Figure 5D), indicating MCM2-7 proteins are also required for inducible recruitment of p65 to κB sites. Rescue of MCM4 partially restored the TNFα-induced recruitment of p65 to the CCL20, CXCL3, IL1A, and PTGS2 promoters in MCM4^KO^ cells, respectively (Supplementary Figure S7), indicating that the recruitment defect is directly attributable to MCM2-7 deficiency. These reciprocal dependency relationships demonstrate that MCM2-7 proteins and p65 exhibit mutually cooperative binding to NF-κB target gene promoters.

### MCM2-7 proteins facilitate RNA Pol II recruitment to NF-κB target genes

**Figure 6:**
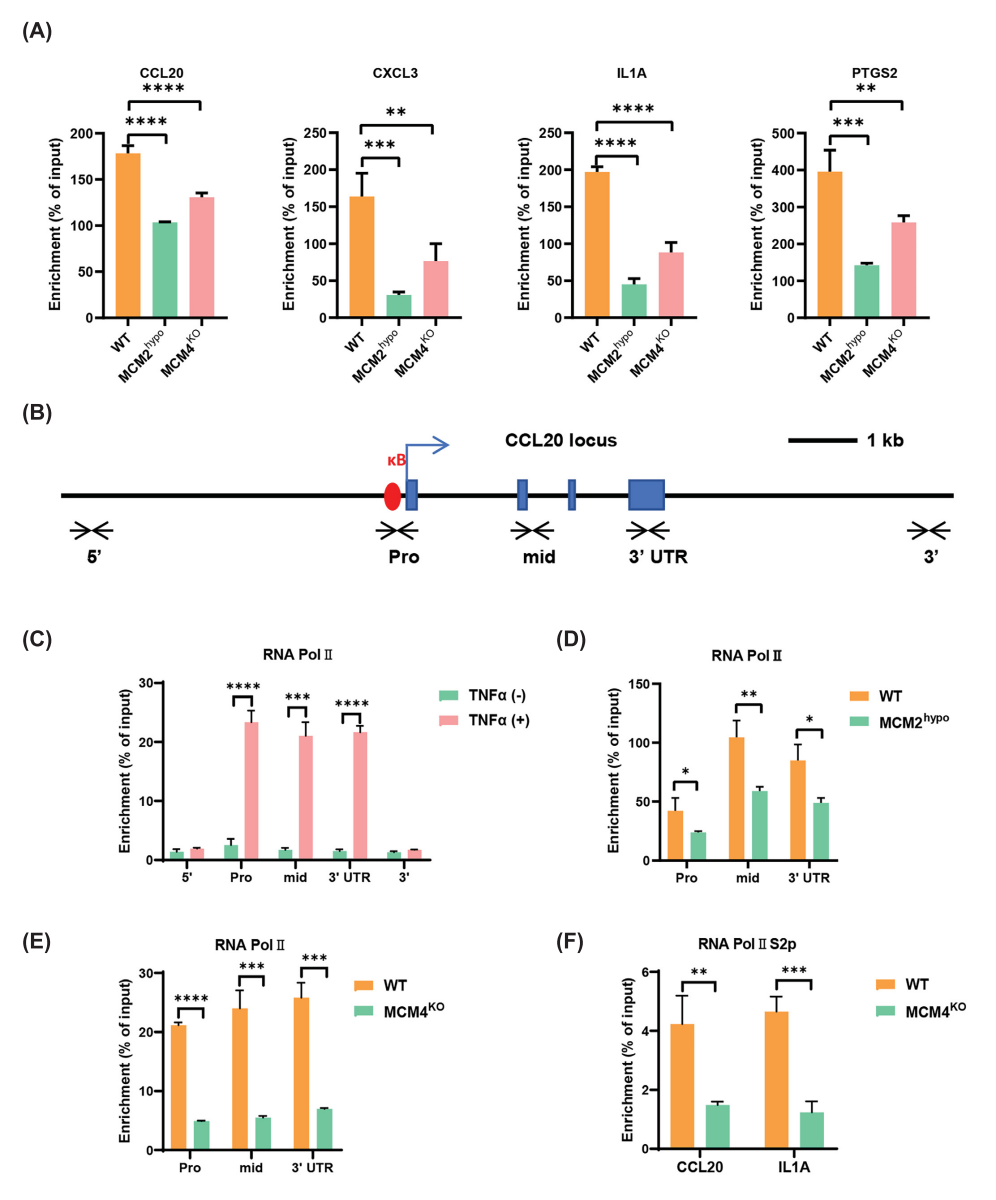
Loss of MCM2-7 proteins attenuate the recruitment of RNA Pol II to NF-κB target gene (**A**) MCM2^hypo^ and MCM4^KO^ as well as WT HeLa cells were treated with TNFα for 30 min followed by ChIP assays with anti-RNA Pol II antibody. Data are presented as mean ± SD. One-way ANOVA with Dunnett’s multiple comparisons test was performed among WT, MCM2^hypo^ and MCM4^KO^. **P<0.01; ***P<0.001; ****P<0.0001. (**B**) The CCL20 locus and the ChIP primer sets. Exons are indicated by blue rectangles, and red oval indicates NF-κB binding site (κB site). Paired arrows present the position of ChIP primer sets. 5′ and 3′, intergenic region 5′ or 3′ of CCL20 locus; Pro, promoter; mid, middle region of gene body; 3′ UTR, 3′ untranslated region. (**C**) HeLa cells were treated with or without TNFα for 30 min, and different regions of CCL20 locus were analyzed by ChIP assays with the anti-RNA Pol II antibodies. Data are presented as mean ± SD. Unpaired *t*-test was performed between treated and untreated groups. ***P<0.001; ****P<0.0001. MCM2^hypo^ (**D**) or MCM4^KO^ (**E**) and WT HeLa cells were treated with TNFα for 30 min, and then CCL20 locus was tested by ChIP assays with anti- RNA Pol II antibody. Data are presented as mean ± SD. Unpaired *t*-test was performed between WT and KO groups. *P<0.05; **P<0.01; ***P<0.001; ****P<0.0001. (**F**) MCM4^KO^ and WT HeLa cells were treated with TNFα for 30 min followed by ChIP assays with anti- RNA Pol II S2p antibody. Data are presented as mean ± SD. Unpaired *t*-test was performed between WT and MCM4^KO^ groups. **P<0.01; ***P<0.001. *n* = 3 biological replicates.

Effective recruitment of RNA Pol II to NF-κB target genes requires prior binding of NF-κB to cognate promoter elements, establishing a sequential recruitment cascade essential for transcriptional activation. The role of MCM2-7 proteins in promoting p65 recruitment to target gene promoters prompted us to examine its potential regulation of RNA Pol II recruitment. Depletion of either MCM2 or MCM4 significantly reduced RNA Pol II binding to κB sites upon TNFα stimulation (Figure 6A), suggesting that MCM2-7 proteins facilitate RNA Pol II recruitment. Consistent with this observation, reconstitution with MCM4 restored TNFα-induced RNA Pol II occupancy at κB sites in MCM4^KO^ cells (Supplementary Figure S8). These findings support the conclusion that MCM2-7 facilitates the assembly of the transcription machinery at NF-κB target genes. To further support the role of the MCM2-7 proteins in NF-κB-mediated transcription, we designed a series of ChIP primers covering the CCL20 locus and analyzed the recruitment of RNA Pol II across the CCL20 locus by ChIP in HeLa cells (Figure 6B). In unstimulated cells, RNA Pol II occupancy at CCL20 was minimal (Figure 6C). Following TNFα stimulation, a significant increase in RNA Pol II occupancy was observed specifically at the promoter, middle, and 3′ UTR regions of CCL20, but not in flanking intergenic regions (Figure 6C). Notably, depletion of MCM2 or MCM4 markedly reduced the TNFα-induced occupancy of RNA Pol II at the promoter, middle and 3′ UTR regions of CCL20 (Figure 6D,E). Consistent with this reduction in RNA Pol II recruitment, MCM4^KO^ also impaired the TNFα-induced occupancy of Ser2-phosphorylated RNA Pol II (Pol II S2p), a mark associated with transcription elongation, at the middle regions of both CCL20 and IL1A genes (Figure 6F). Together, these results support a model in which MCM2-7 proteins enhance the assembly of the transcription pre-initiation complex at NF-κB target promoters by facilitating p65 and RNA Pol II recruitment, thereby promoting transcriptional activation. The reduced RNA Pol II occupancy across gene bodies in MCM-deficient cells further underscores the importance of MCM2-7 in sustaining efficient transcription of NF-κB-regulated genes.

## Discussion

**Figure 7:**
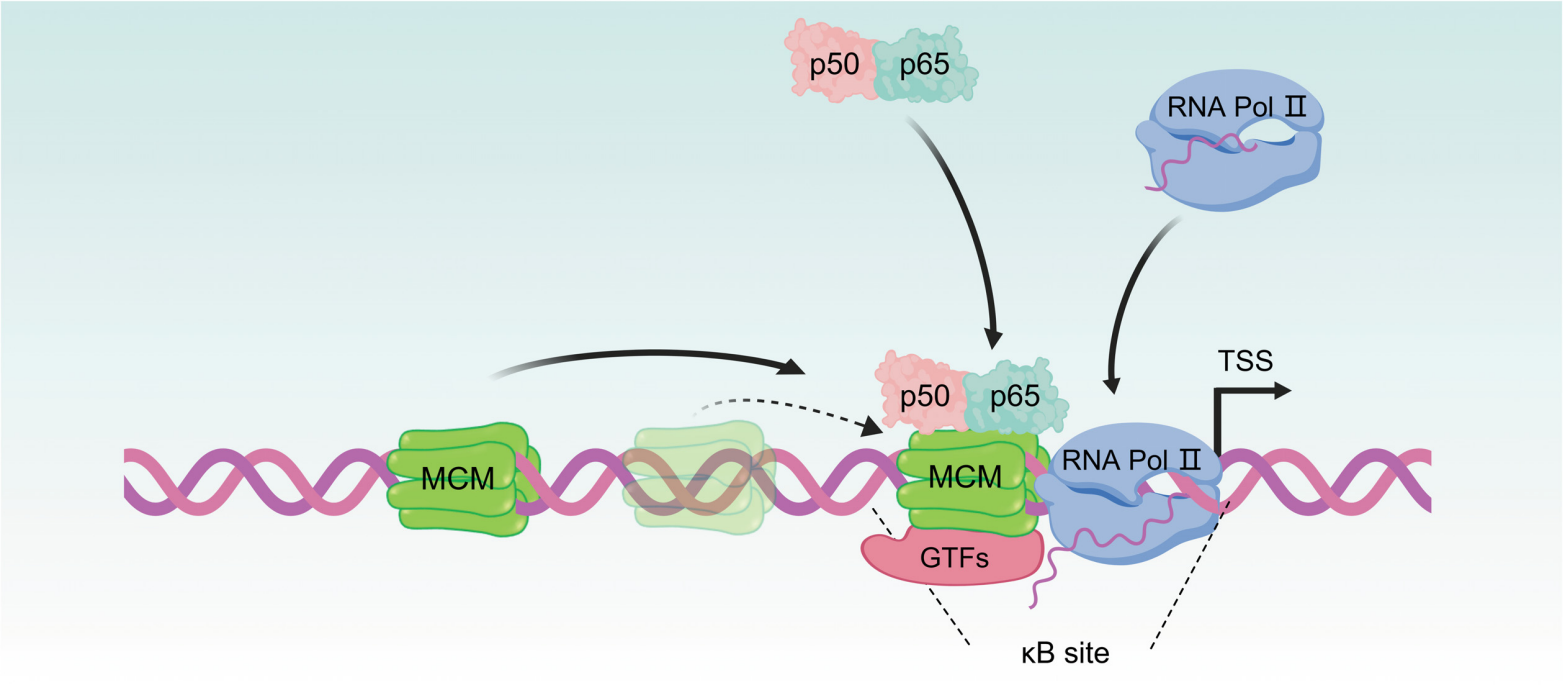
Model for the role of MCM2-7 complex in NF-κB-dependent gene regulation

In the present study, we show that MCM2-7 proteins are involved in NF-κB activation and transcription of its target genes. Mechanistically, MCM2-7 proteins interact with NF-κB p65 and promote the formation of transcription pre-initiation complex comprised RNA Pol II and p65 on the promoters of NF-κB targets (Figure 7). We also demonstrated genetic ablation of MCM2-7 components significantly reduces RNA Pol II occupancy and Pol II S2p levels throughout the transcribed regions. These observations reveal a previously unidentified functional role for MCM2-7 proteins in regulating NF-κB target gene transcription.

RNA Pol II and a set of general transcription factors (GTFs) assemble into a transcription pre-initiation complex at the core promotor of active genes, which is a critical rate-limiting step in transcriptional activation [24]. Nuclear p65-p50 dimer binds to κB sites in the promoter or enhancer regions of target genes, and then interacts with coactivators such as CBP/P300 and Mediator complex to help recruit RNA Pol II and GTFs [25,26]. Our data show that multiple components of MCM2-7 complex interact with p65 and are recruited to the κB site-containing DNA regions of NF-κB targets by p65. Furthermore, MCM2-7 proteins in turn facilitate the formation of the transcription pre-initiation complex by the recruitment of p65 and RNA Pol II at the promoters of NF-κB target genes. Consistent with the obligate heterohexameric nature of the MCM2-7 complex, we observed that depletion of a single subunit leads to reduced steady-state levels of the remaining components (Figures 2A and 3A,C). This interdependence aligns with the finding that unassembled orphan MCM subunits are selectively recognized and degraded via cellular protein quality control pathways [27]. Thus, the transcriptional defects we observe stem from compromised MCM2-7 complex function rather than isolated subunit activities. Interestingly, the previous observations demonstrated that MCM2-7 proteins copurify with RNA Pol II and GTFs [28], and MCM2 associates with RNA Pol II and GTFs [29]. These data suggest that MCM2-7 complex might serve as a transcriptional coactivator to promote the assembly of the transcription pre-initiation complex at NF-κB target promoters through the interaction with RNA Pol II and GTFs, following rapid and specific activation of target gene expression upon induction. In addition, our previous reports indicated that FAM21 interacts with p65 and contributes to p65 loading on NF-κB-responsive chromatin regions [15], and FAM21 also associates with MCM2-7 proteins [16]. Therefore, these findings raise the possibility that MCM2-7 proteins and FAM21 might associate into a complex to modulate p65 chromatin loading as well as assembly of transcription pre-initiation complex.

It has been reported that MCM2-7 proteins are involved in the modulation of inducible gene expression through the interaction with transcription factors. MCM5 directly interacts with STAT1 and is essential for STAT1-mediated transcriptional activation in response to IFNγ [8]. Importantly, WT MCM5 enhances STAT1 activity, whereas MCM5 mutants deficient for helicase activity and MCM2-7 complex assembly fail to potentiate STAT1-dependent transcriptional activation, indicating that intact MCM2-7 complex and its helicase activity are required for STAT1 transactivation [8]. It is worth noting that the MCM2-7 proteins are recruited to STAT1 target gene promoters and move along the target gene body with the RNA Pol II during transcription elongation [9]. Interestingly, an earlier study showed that MCM2-7 complex moves ahead of elongating RNA polymerase along the chromatin in budding yeast, although it did not address whether MCM2-7 complex is required for RNA polymerase-mediated transcription [30]. Similarly, we observed that MCM2-7 complex is recruited to the NF-κB target gene body, and loss of MCM proteins reduces the occupancy of RNA Pol II on the intragenic regions of NF-κB target gene. Collectively, these observations suggest that MCM2-7 complex might be necessary for optimal RNA Pol II-dependent elongation. However, in contrast to these effects on transcriptional activation, MCM2-7 proteins are recognized as negative regulators of HIF-1 [10]. Both MCM3 and MCM7 directly interact with HIF-1, and they inhibit HIF-1 activity in response to hypoxia. MCM3 inhibits HIF-1 transactivation domain function, while MCM7 enhances HIF-1 ubiquitination and proteasomal degradation [10]. Our results demonstrated that MCM2-7 complex interacts with p65 to facilitate NF-κB transcriptional activation but does not affect p65 stability. Therefore, MCM2-7 proteins interact with transcription factors to regulate inducible gene transcription through distinct molecular mechanisms.

Transcription factors bind to enhancers of target genes and mediate enhancer–promoter interactions [31], which helps recruit RNA Pol II through the interaction with mediator complex and GTFs followed by specific gene transcription [32]. Notably, cohesin complex, viewed as a key regulator of 3-dimensional (3D) genome organization, promotes the formation of enhancer–promoter loops by bring enhancers into close physical proximity with the target gene promoters, triggering transcriptional activation [33]. Interestingly, in addition to the effect on RNA Pol II initiation at promoters, cohesin was observed to affect RNA Pol II elongation in the gene body [34]. Moreover, cohesin was reported to control the expression of LPS-induced inflammatory genes involved in TNFα signaling pathway in mouse macrophages [35]. Importantly, there is now increasing evidence that MCM2-7 complex is a key regulator of cohesin activity [36]. A recent study indicated that MCM2-7 complex modulates cohesin-mediated DNA loop formation [37]. Together with our findings that MCM2-7 proteins function on NF-κB activity, these data suggest that MCM2-7 complex might work with cohesin to regulate inducible gene expression in response to inflammatory stimuli via the organization of 3D chromatin structure.

We also note that MCM4^KO^ cells, which carry a complete loss-of-function mutation in an essential replication gene, may have acquired compensatory mutations during prolonged culture. Although we cannot formally exclude this possibility, the functional rescue experiments argue against a major contribution of such secondary alterations to the NF-κB transcriptional phenotypes. Re-expression of full-length MCM4 in MCM4^KO^ cells partially restored both target gene expression and promoter recruitment of p65 and RNA Pol II. If the observed defects were driven by compensatory mutations affecting NF-κB signaling independently of MCM4 loss, re-expression of MCM4 would not be expected to reverse the phenotypes. The rescue data therefore support the interpretation that the transcriptional impairments are a direct consequence of MCM2-7 complex dysfunction.

Several limitations of this study should be noted. First, although BrdU incorporation assays revealed no gross alterations in S-phase progression in shMCM2, shMCM6, MCM2^hypo^, and MCM4^KO^ cells (Supplementary Figure S3), this approach cannot fully exclude the possibility that subtle replication stress or localized fork slowing contributes indirectly to the observed transcriptional changes. Because MCM2-7 is essential for replication licensing, constitutive depletion models are inherently limited in their ability to resolve replication-dependent and replication-independent functions. Formal demonstration of a replication-independent role will require acute depletion approaches, such as auxin-inducible degron alleles in G1-arrested cells, which bypass the confounding effects of replication licensing. Until such experiments are available, an indirect contribution of replication perturbations cannot be entirely ruled out. Second, while the ΔAAA mutant data suggest that the MCM4 AAA+ domain is important for transcriptional activity, deletion of the entire domain cannot distinguish between a requirement for helicase catalysis and a structural or scaffolding role. Addressing this question will require Walker motif point mutations that selectively disable ATP hydrolysis without removing the domain.

In summary, the present study demonstrates that MCM2-7 proteins interact with p65 to regulate NF-κB activity by facilitating the assembly of transcription pre-initiation complex at promoters. Nonetheless, whether the helicase activities of MCM2-7 proteins required for NF-κB transcriptional regulation need to be further investigated.

## Supplementary Material

Supplementary Figures S1-S9 and Tables S1-S3

## Data Availability

The raw sequence data reported in this paper have been deposited in the Genome Sequence Archive [38] of National Genomics Data Center [39] under accession number HRA012095, and are publicly accessible at https://ngdc.cncb.ac.cn/gsa-human. Further inquiries can be directed to the corresponding authors.

## Abbreviations

ANOVA: analysis of variance
CDK: cyclin-dependent kinase
ChIP: chromatin immunoprecipitation
DEG: differentially expressed gene
GSEA: gene set enrichment analysis
GTF: general transcription factor
HIF-1: hypoxia-inducible factor 1
IκB: inhibitor of κB
LPS: lipopolysaccharide
MCM4^KO^: MCM4 knockout
MCM2-7: minichromosome maintenance 2-7
NF-κB: nuclear factor κB
PI: propidium iodide
PLA: proximity ligation assay
Poll II: polymerase II
qRT-PCR: quantitative real-time PCR
SD: standard deviation
siRNA: small interfering RNA
STAT1: signal transducer and activator of transcription 1
TNFα: tumor necrosis factor alpha
WT: wild type
