# Supplementary material for: MCM2-7 proteins promote NF-κB transcriptional activity through cooperative promoter recruitment
Source: Biosci Rep. 2026 Jun 24;46(7):BSR20260337. doi: 10.1042/BSR20260337 (PMC13305959; doi:10.1042/BSR20260337)
Supplement: Supplementary Figures S1-S9 and Tables S1-S3 [file BSR-2026-0337_supp.pdf]

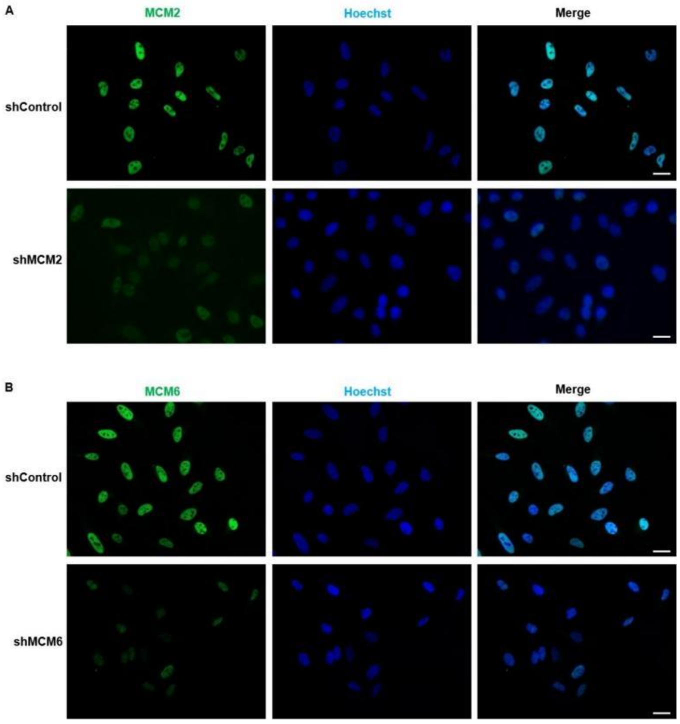

Figure S1. Validation of MCM2 and MCM6 antibody specificity in HeLa cells. (A) Immunofluorescence staining of MCM2 (green) in shControl versus shMCM2 HeLa cells. (B) Immunofluorescence staining of MCM6 (green) in shControl versus shMCM6 HeLa cells. Nuclei were counterstained with Hoechst (blue). Scale bars: 20 μm.

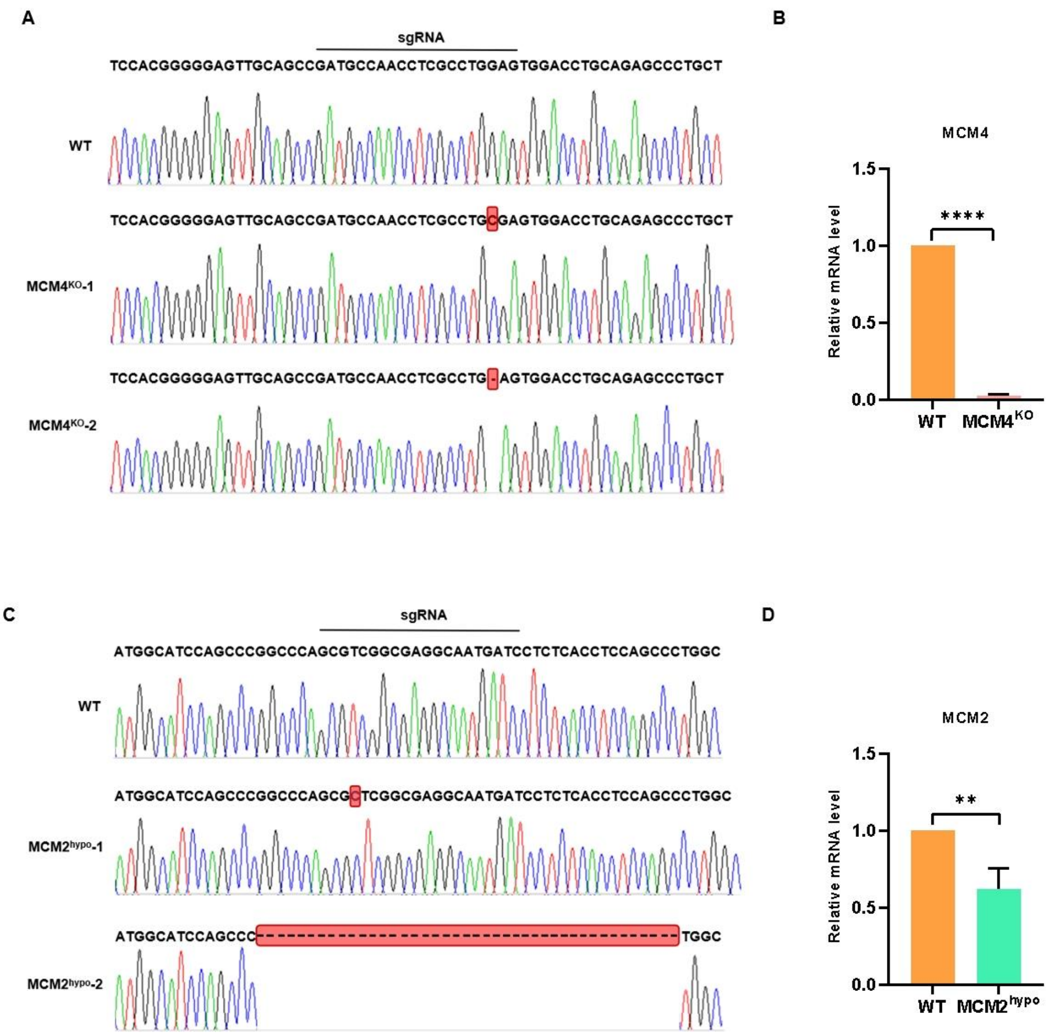

Figure S2. Validation of CRISPR-Cas9-mediated genome editing in MCM4 and MCM2. (A) Sanger sequencing analysis of MCM4 genomic editing. PCR amplicons flanking the sgRNA target site were cloned into the pCDNA3.1 vector, and five individual subclones were subjected to Sanger sequencing. Two distinct frameshift mutation patterns were identified: a single-base insertion and a single-base deletion. (B) qRT-PCR analysis of MCM4 mRNA levels in WT and MCM4^{KO} cells. Data are presented as mean ± SD. ****, $p < 0.0001$. Unpaired t test was performed between WT and mutant cells. n = 3 biological replicates. (C) Sanger sequencing analysis of MCM2 genomic editing. PCR amplicons flanking the sgRNA target site were cloned into the pCDNA3.1 vector, and seven individual subclones were subjected to Sanger sequencing. Two mutation patterns were detected: a single-base insertion resulting in a frameshift, and a 42-bp in-frame deletion. (D) qRT-PCR analysis of MCM2 mRNA levels in WT and MCM2^{hypo} cells. Data are presented as mean ± SD. **, $p < 0.01$. Unpaired t test was performed between WT and mutant cells. n = 3 biological replicates.

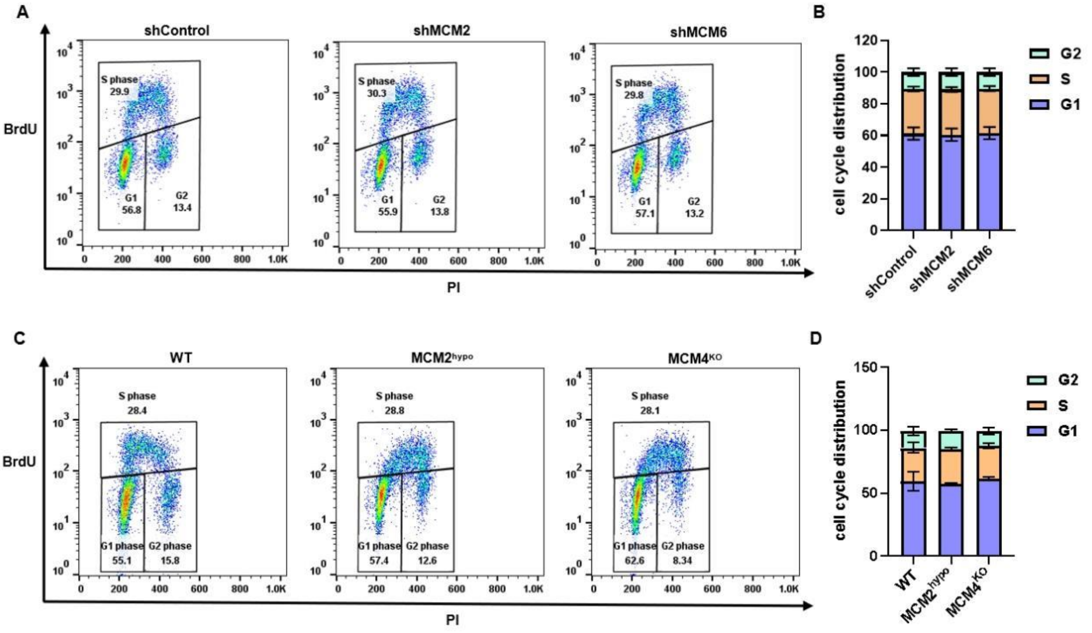

Figure S3. MCM depletion does not alter cell cycle progression. (A) Cell cycle analysis by flow cytometry in shControl, shMCM2, and shMCM6 HeLa cells using BrdU incorporation (anti-BrdU antibody) combined with PI staining. (B) Quantified cell cycle phase distributions (G1/S/G2) showing comparable progression among shControl, shMCM2 and shMCM6 HeLa cells. (C) Cell cycle analysis by flow cytometry in WT, MCM2^{hypo} and MCM4^{KO} HeLa cells using BrdU incorporation combined with PI staining. (D) Quantified cell cycle phase distributions (G1/S/G2) showing comparable progression among WT, MCM2^{hypo} and MCM4^{KO} HeLa cells.

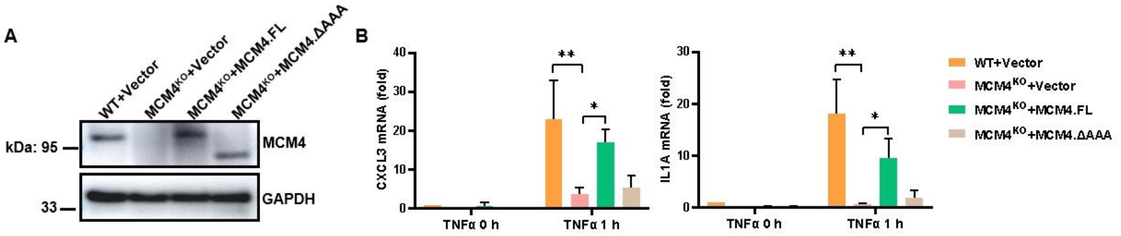

Figure S4. AAA+ domain of MCM4 is required for TNFα-induced mRNA expression of selected NF-κB targets. (A) Expression of MCM4 was examined by western blotting in HeLa stable cells. (B) qRT-PCR was performed to analyze the basal and TNFα-induced mRNA expression of the indicated NF-κB target genes. Data are presented as mean ± SD. *, $p < 0.05$; **, $p < 0.01$. One-way ANOVA with Dunnett's multiple comparisons test was performed among WT+Vector, MCM4^{KO}+Vector, MCM4^{KO}+MCM4.FL and MCM4^{KO}+MCM4.ΔAAA. n = 3 biological replicates.

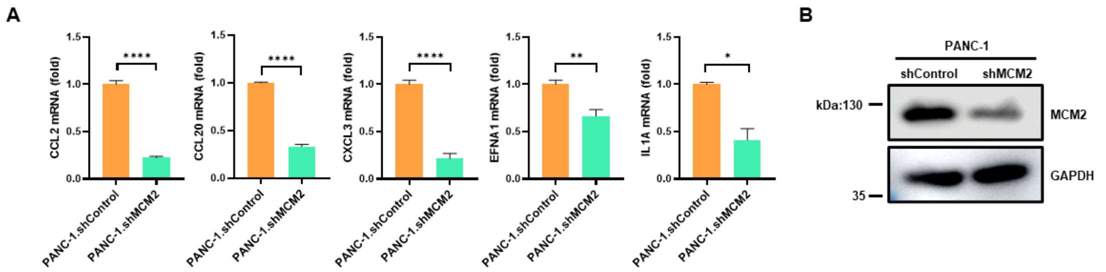

Figure S5. MCM2 depletion reduces transcription of NF-κB target genes in PANC-1 cells. (A) The basal mRNA expression of selected NF-κB targets was analyzed by qRT-PCR in PANC-1 stable cells. (B) Expression of MCM2 was examined by western blotting in PANC-1 stable cell lines. Data are presented as mean ± SD. *, $p < 0.05$; **, $p < 0.01$; **** $p < 0.0001$. Unpaired t test was performed between shControl and shMCM2. n = 3 biological replicates.

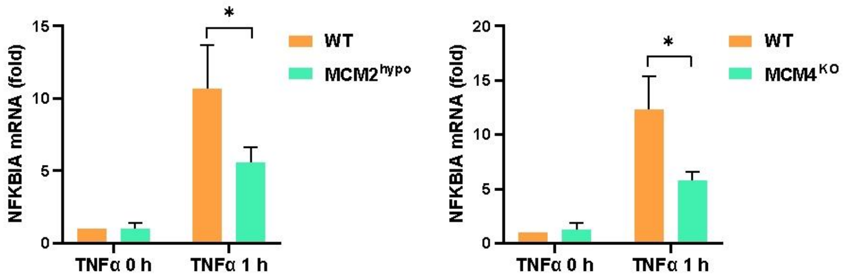

Figure S6. MCM depletion decreases NFKBIA transcript levels in HeLa cells. qRT-PCR analysis of NFKBIA mRNA levels in the indicated cells. Data are presented as mean ± SD. *, $p < 0.05$. Unpaired t test was performed between WT and MCM-depleted cells. n = 3 biological replicates.

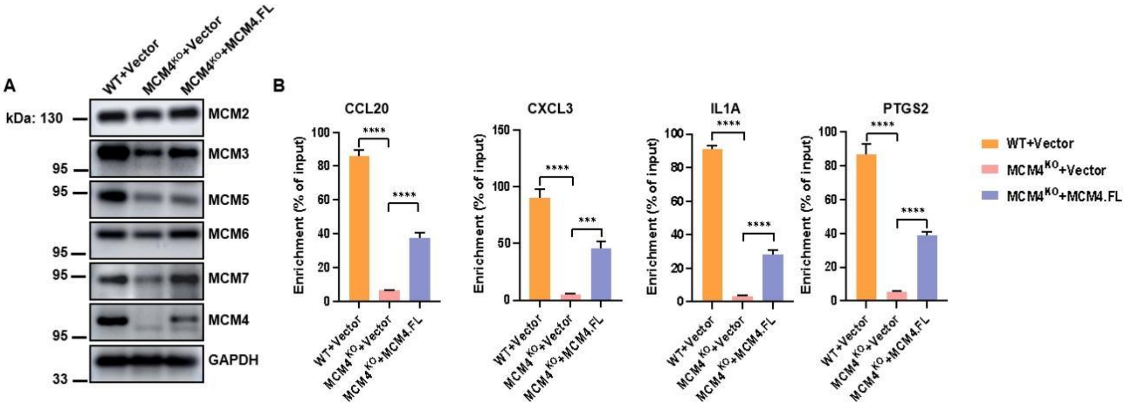

Figure S7. Reconstitution with MCM4 restored TNFα-induced p65 occupancy at κB sites in $MCM4^{KO}$ cells. (A) Expression of the indicated MCM2-7 subunits were examined by western blotting in HeLa stable cells. (B) MCM4 restored TNFα-induced p65 occupancy at κB sites in $MCM4^{KO}$ cells. WT+Vector, $MCM4^{KO}$+Vector, and $MCM4^{KO}$+MCM4.FL HeLa cells were treated with TNFα for 30 min followed by ChIP assays with anti-p65 antibody. Data are presented as mean ± SD. ***, $p < 0.001$; ****, $p < 0.0001$. n = 3 biological replicates.

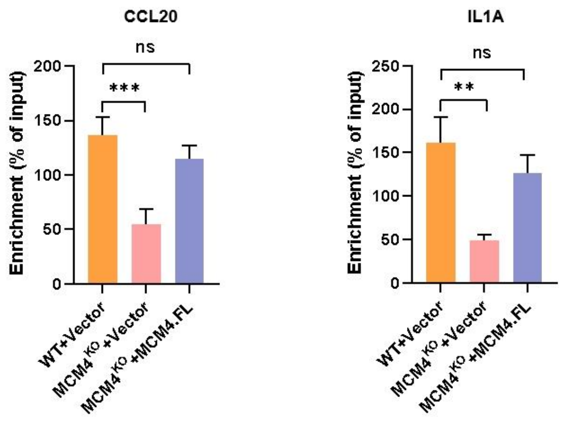

Figure S8. Reconstitution with MCM4 restored TNFα-induced RNA Pol II occupancy at κB sites in MCM4^{KO} cells. WT+Vector, MCM4^{KO}+Vector, and MCM4^{KO}+MCM4.FL HeLa cells were treated with TNFα for 30 min followed by ChIP assays with anti-RNA Pol II antibody. Data are presented as mean ±SD. **, $p < 0.01$; ***, $p < 0.001$; ns, not significant. n = 3 biological replicates.

Table S1. Primers used for the plasmids construction

Primers	Sequence (5'-3')
MCM2-Indel-F	ccaagctggctagttAAGCTTTCTGGAGGCTGGAAGGGGTTT
MCM2-Indel-R	gtgctggatatctgcaGAATTCCTGGGGTGCTTATGGAGTCGG
MCM4-Indel-F	ccaagctggctagttAAGCTTGTGAGTCATAATGCCCCAAGGAA
MCM4-Indel-R	gtgctggatatctgcaGAATTCTAGGAAATTCTGGTGGCATATCAAAC
MCM4.FL-F	ggatgacgacgataagGGATCCATGTCGTCCCCGGCGTCGAC
MCM4.FL-R	taaccggtacgcgtcaCTCGAGTCAGAGCAAGCGCACGGTCTTCC
MCM4. △ AAA-N-R	gtctggtttcctggaAAGTTCCTTAAGCAA
MCM4.ΔAAA-C-F	tccaggaaaccagac GCACTGTACTACCAGAGCGAGGAGCAGG

In primer sequences, lowercase letters indicate homologous arms to the pCDNA3.1 or pLenti6.3 vector for seamless cloning. F and R in primer names denote forward and reverse primers, respectively. Primers containing 'Indel' are used to assess the genomic editing status of the corresponding genes; their amplicons are inserted into the pCDNA3.1 vector via homologous recombination. Primers containing 'FL' anneal to the ends of the MCM4 coding sequence, and those containing 'ΔAAA' target its internal region. The pair MCM4.FL-F/MCM4.FL-R amplifies full-length MCM4; MCM4.FL-F/MCM4.ΔAAA-N-R amplifies the N-terminal fragment; and MCM4.ΔAAA-C-F/MCM4.FL-R amplifies the C-terminal fragment. These amplicons are inserted into the pLenti6.3 vector by homologous recombination.

Table S2. Primer sequences used for qRT-PCR

Primers	Sequence (5'-3')
CCL2-F	CCTCCAGCATGAAAGTCTCTG
CCL2-R	TCTGCACTGAGATCTTCCTATTG
CCL20-F	TGCTGTACCAAGAGTTTGCTC
CCL20-R	CGCACACAGACAACTTTTTCTTT

CXCL3-F	CGCCCAAACCGAAGTCATAG
CXCL3-R	GCTCCCCTTGTTCAGTATCTTTT
EFNA1-F	TCAGGCCCATGACAATCCAC
EFNA1-R	GTGACCGATGCTATGTAGAACC
IL-1A-F	TGTATGTGACTGCCCAAGATG
IL-1A-R	TTAGTGCCGTGAGTTTCCC
PTGS2-F	CTGGCGCTCAGCCATACAG
PTGS2-R	CGCACTTATACTGGTCAAATCCC
NFκBp65-F	GTGGGGACTACGACCTGAATG
NFκBp65-R	GGGGCACGATTGTCAAAGATG
NFKBIA-F	CTCCGAGACTTTCGAGGAAATAC
NFKBIA-R	GCCATTGTAGTTGGTAGCCTTCA
MCM2-F	ATGATCGAGAGCATCGAGAACC
MCM2-R	GCCAAGTCCTCATAGTTCACCA
MCM4-F	GACGTAGAGGCGAGGATTCC
MCM4-R	GCTGGGAGTGCCGTATGTC
GAPDH-F	AATGAAGGGGTCATTGATGG
GAPDH-R	AAGGTGAAGGTCGGAGTCAA

F and R in primer names denote forward and reverse primers, respectively.

Table S3. Primer sequences used for CHIP-qPCR

Primers	Sequence (5’-3’)
IL1A.pro-F	TGTCCTGCCTCAGCCTCC
IL1A.pro-R	CACCAGACTCTTAGCAAATGAATG
CXCL3.pro-F	GAGGCGTAGGCGTCACCAG
CXCL3.pro-R	CAAGATCGGCGAACCCTTT
PTGS2.pro-F	GTCAGCCTTTCTTAACCTTACTCG
PTGS2.pro-R	GGGGAGGGCAGAAGGACAC
CCL20.pro-F	CACCCTGACCTTCGCACCT
CCL20.pro-R	AGCCTGGGATGGCCCTAT
CCL20.5Flank-F	TCCTTTCCTTTCTATGCGTTTT
CCL20.5Flank-R	TTGGGTTGGGCAATGACTT
CCL20.Mid-F	CTCCTCCTCTAAGTGGTTTATTCG
CCL20.Mid-R	CAGTCAAAGTTGCTTGCTGCTAA
CCL20.3UTR-F	GCATAGCCCAAGAACAGAAAGAAC
CCL20.3UTR-R	GACAAGTCCAGTGAGGCACAA
CCL20.3Flank-F	CAAAAGGCGGGTTTCCAG
CCL20.3Flank-R	TCAACTTCCTCCATCCCAAAT
IL1A.Mid-F	GAAGCACATAAGCAACAACAAGG
IL1A.Mid-R	GGAAAATAGTTCTGGAGGGGATA

F and R in primer names denote forward and reverse primers, respectively. In primer names, “pro” indicates primers targeting the promoter region of the corresponding gene, “Flank” indicates primers

targeting the intergenic region 5’ or 3’ of the CCL20 locus, “Mid” indicates primers targeting the middle region of the gene body, and “3UTR” indicates primers targeting the 3’ untranslated region.

Supplementary Fig. 1 Original images of PLA shown in Fig.1A

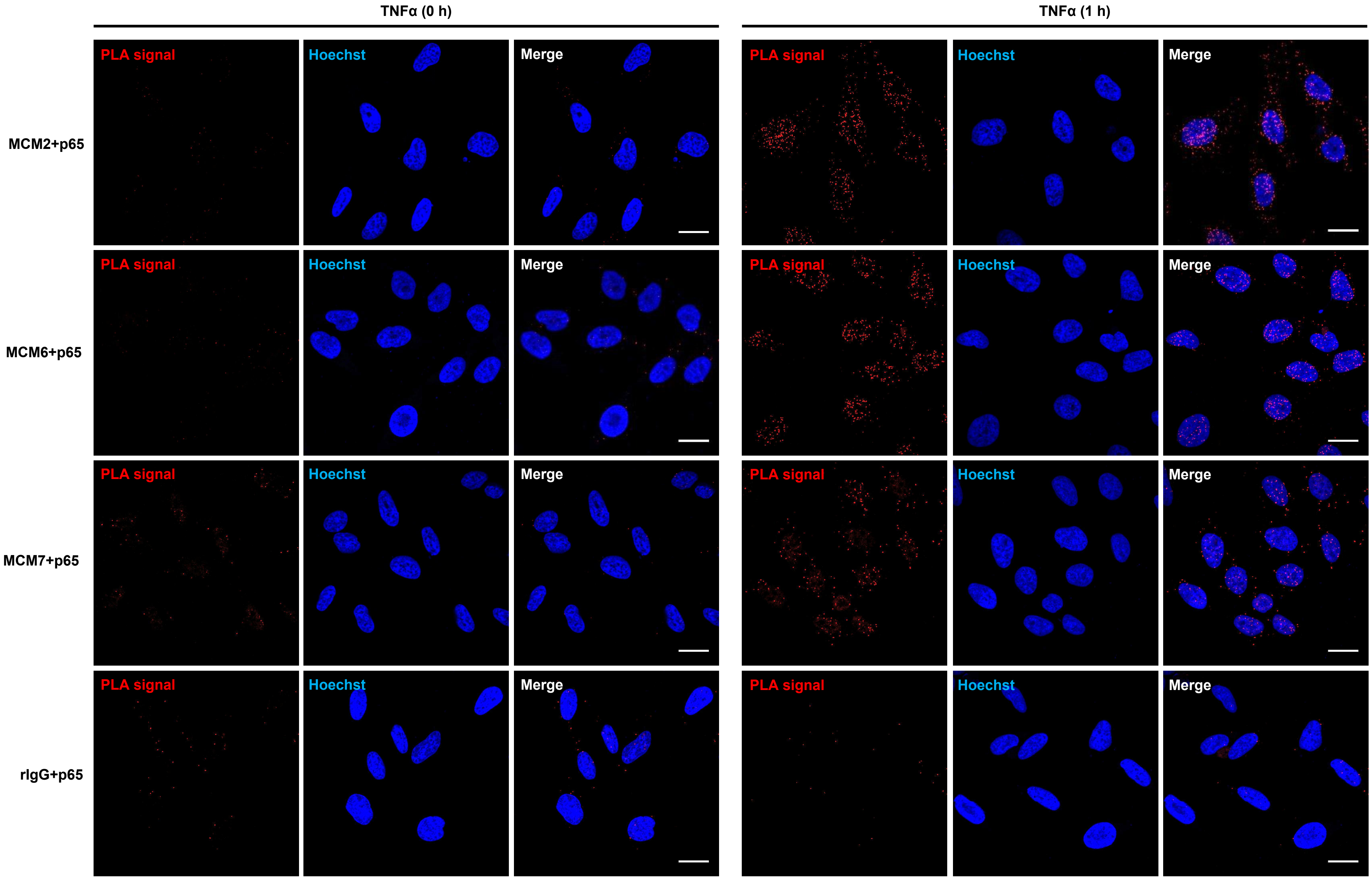

Supplementary Fig. 2 Original images of PLA shown in Fig. 1B

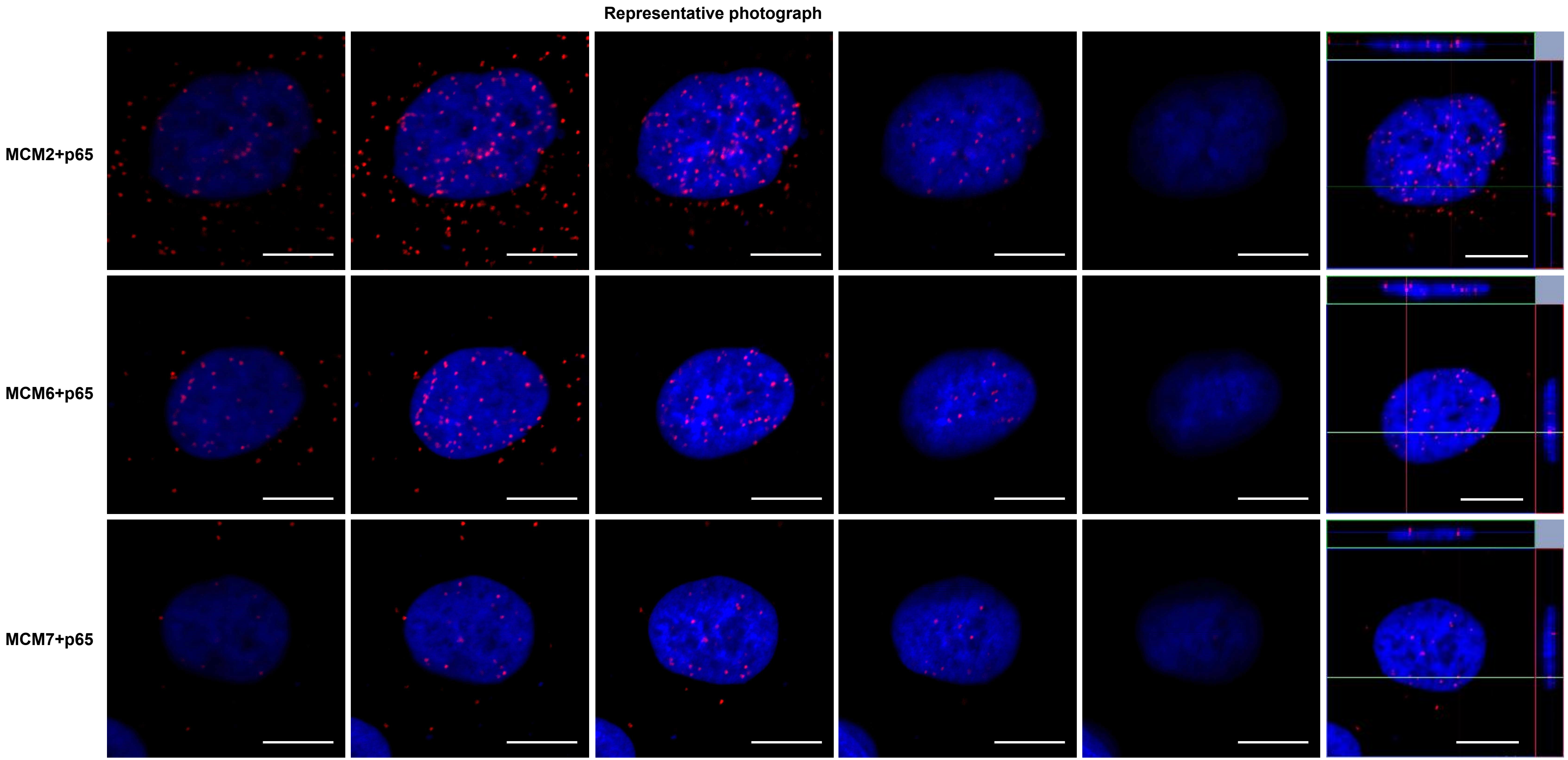

Supplementary Fig. 3 Original images of western blotting shown in Fig. 1D

Number 1 and number 2 are from the same membrane, number 3 and number 4 are from the same membrane.

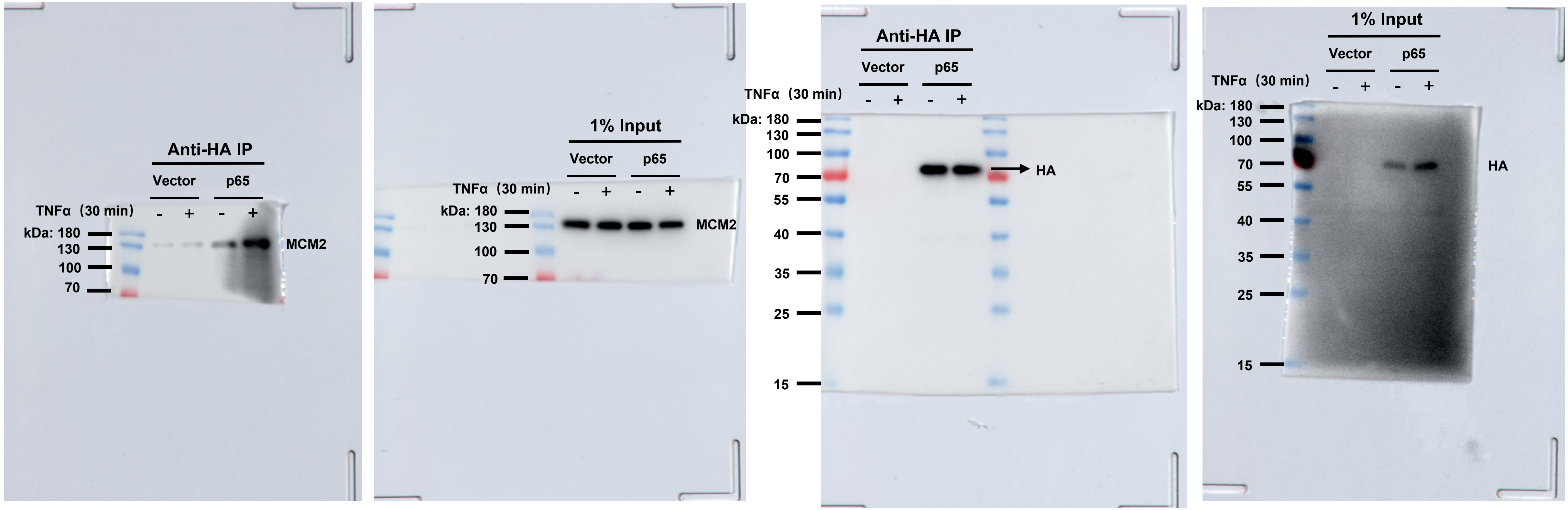

① ② ③ ④

Marker: Thermo 26616

Supplementary Fig. 4 Original images of western blotting shown in Fig. 2A

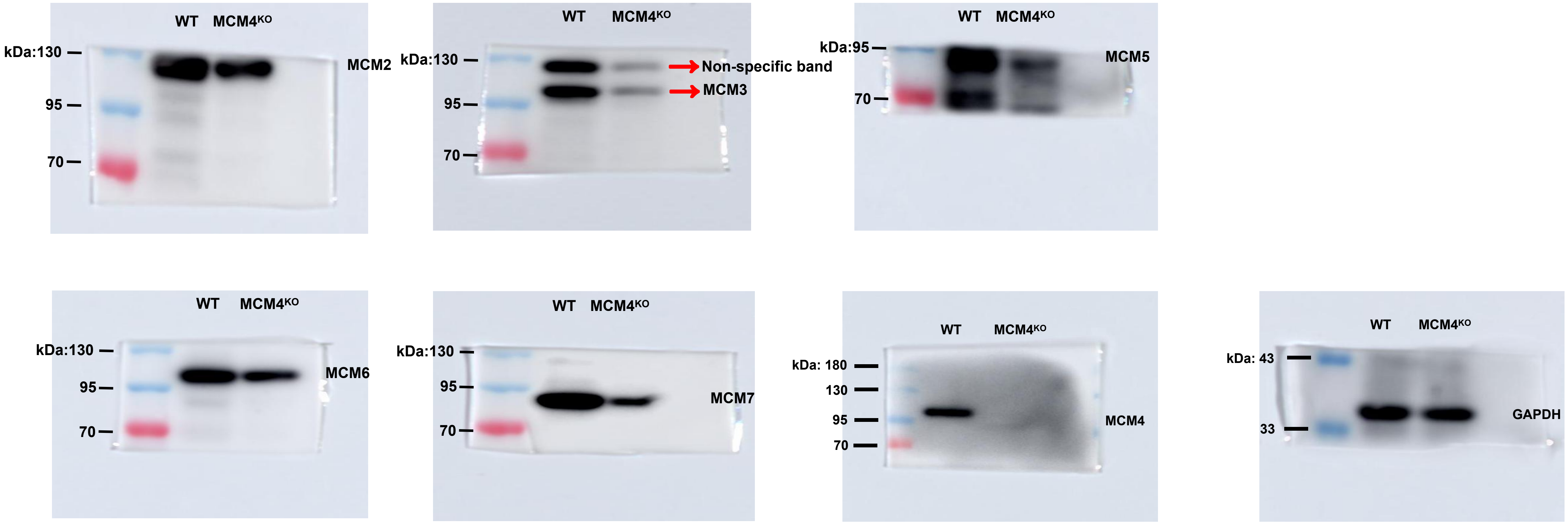

Marker： Thermo 26616

Supplementary Fig. 5 Original images of western blotting shown in Fig. 3A

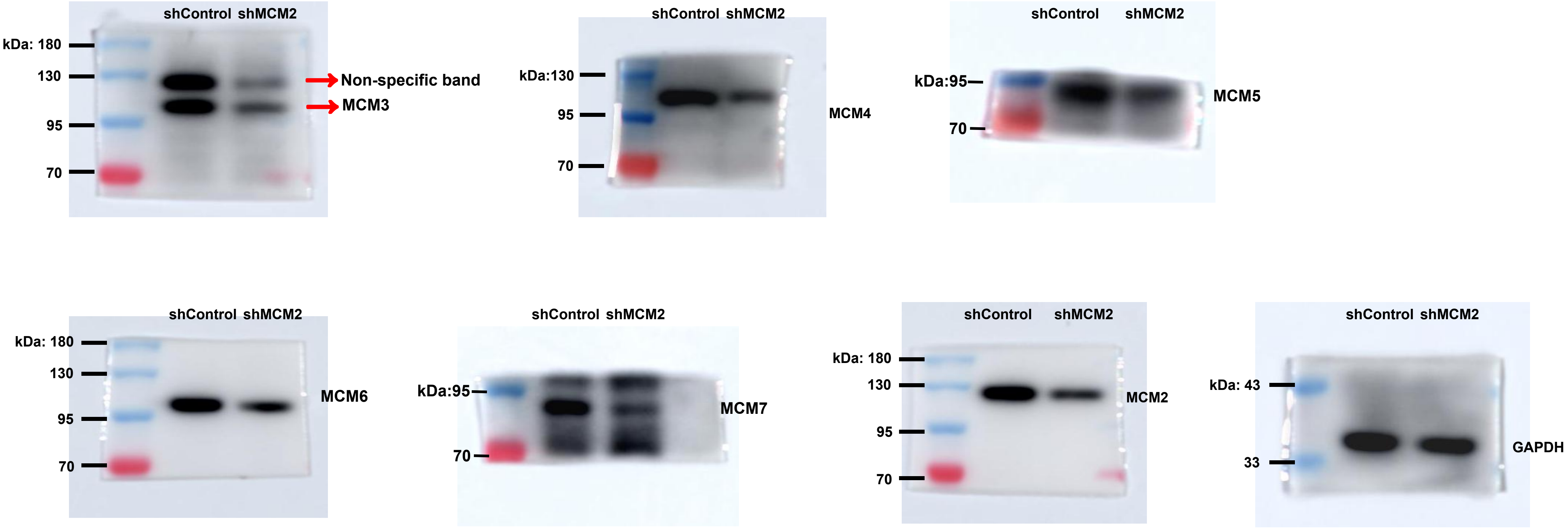

Marker：Biosharp BL712A

Supplementary Fig. 5 Original images of western blotting shown in Fig. 3A

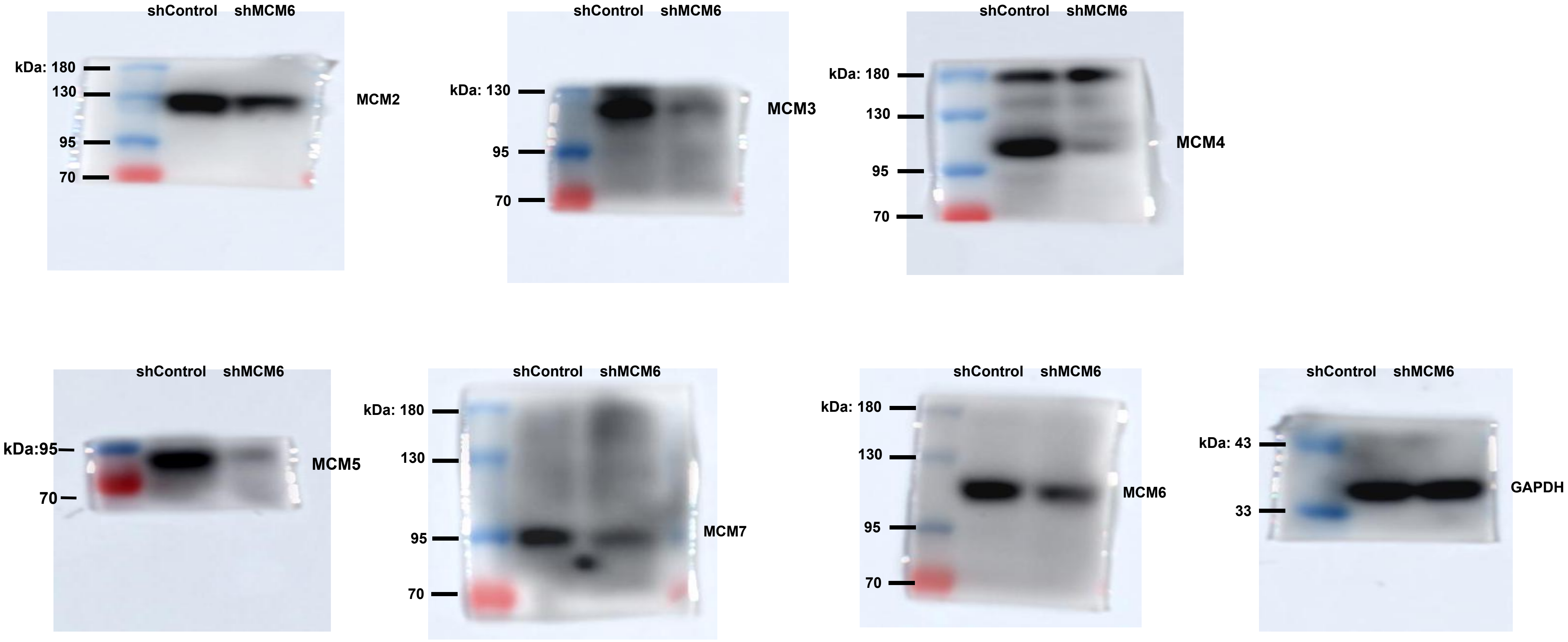

Marker： Biosharp BL712A

Supplementary Fig. 6 Original images of western blotting shown in Fig. 3C

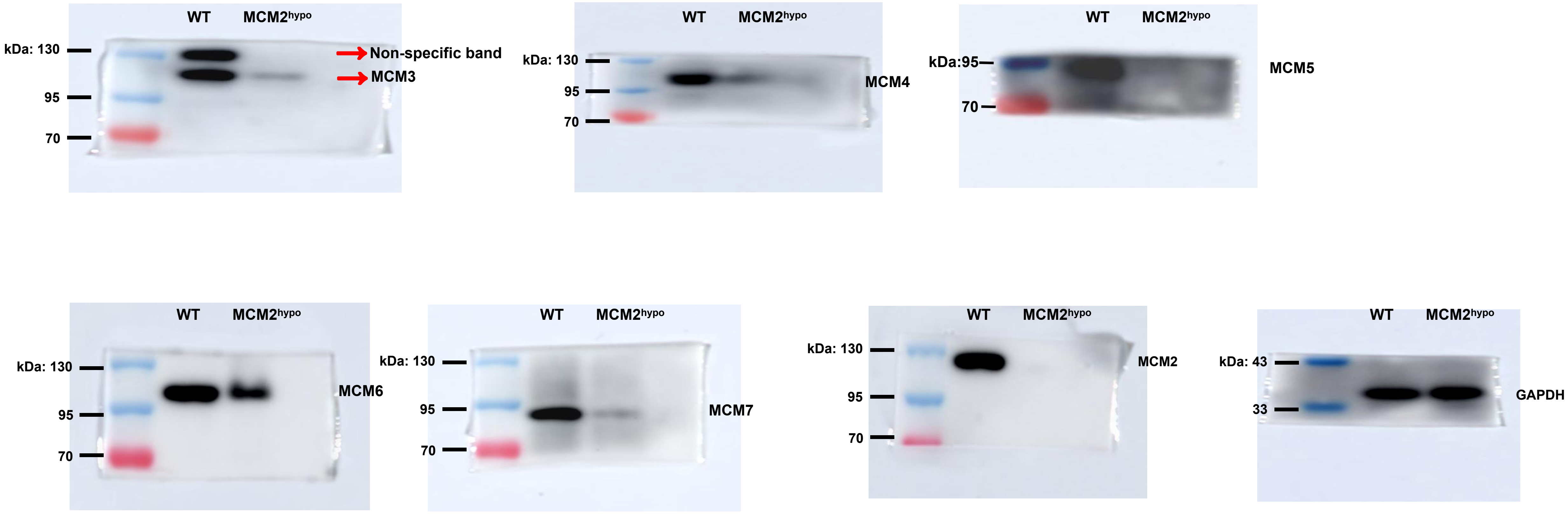

Marker： Biosharp BL712A

Supplementary Fig. 7 Original images of western blotting shown in Fig. 4A

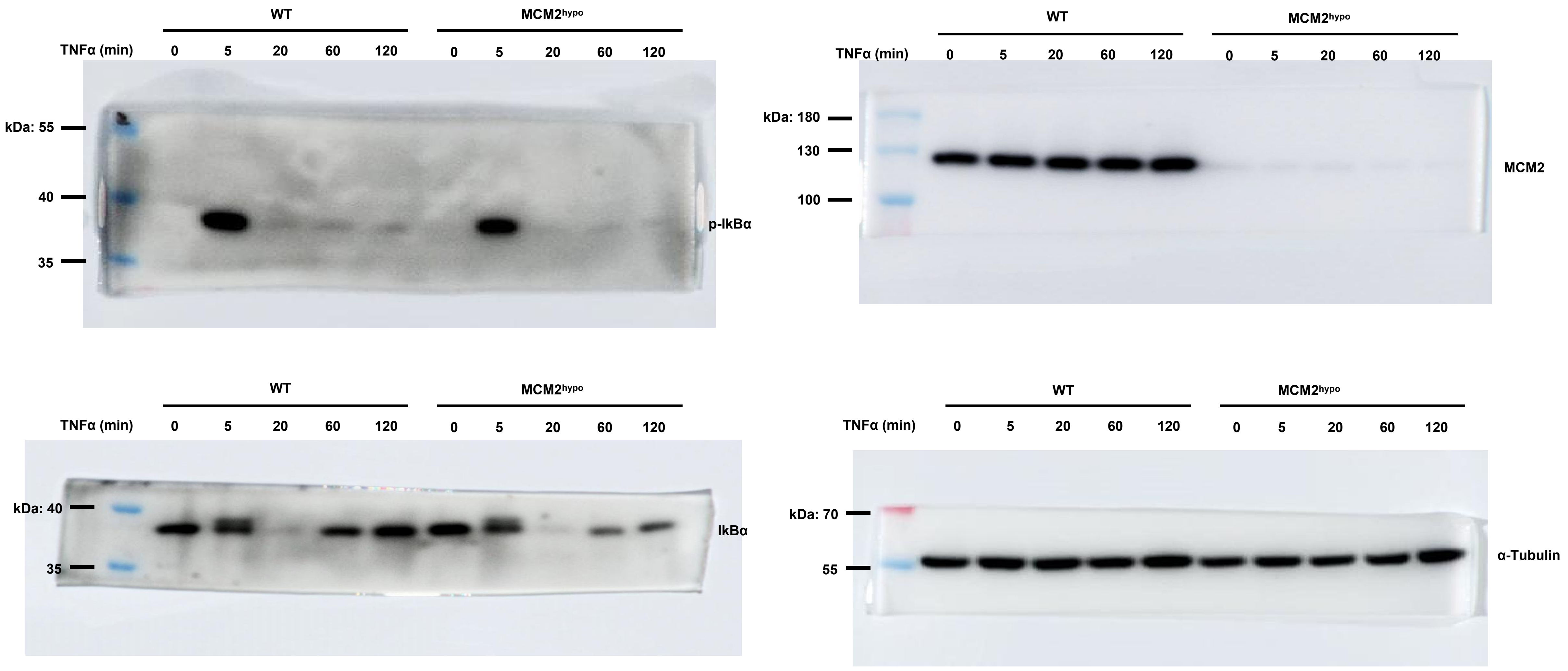

Marker: Thermo 26616

Supplementary Fig. 8 Original images of western blotting shown in Fig. 4B

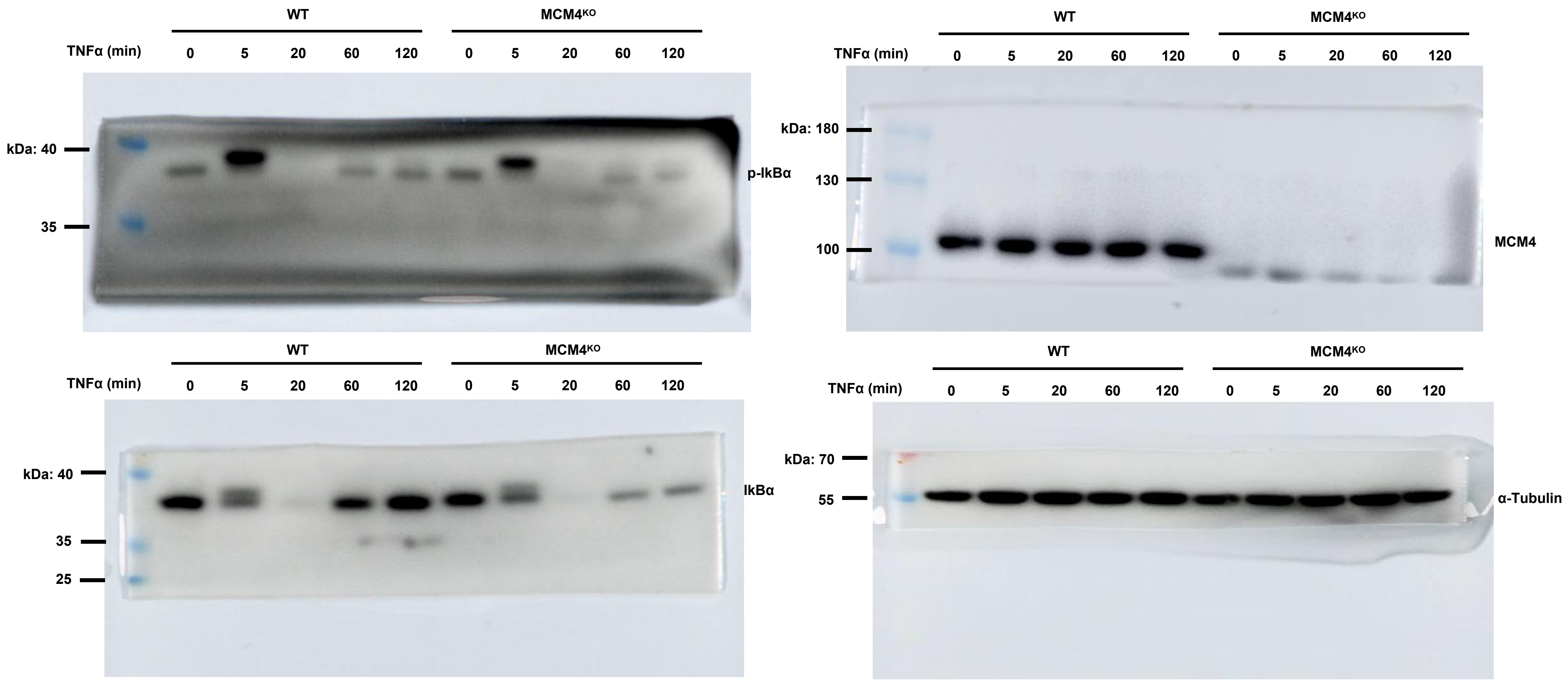

Marker： Thermo 26616

Supplementary Fig. 9 Original images of immunofluorescence shown in Fig. 4C

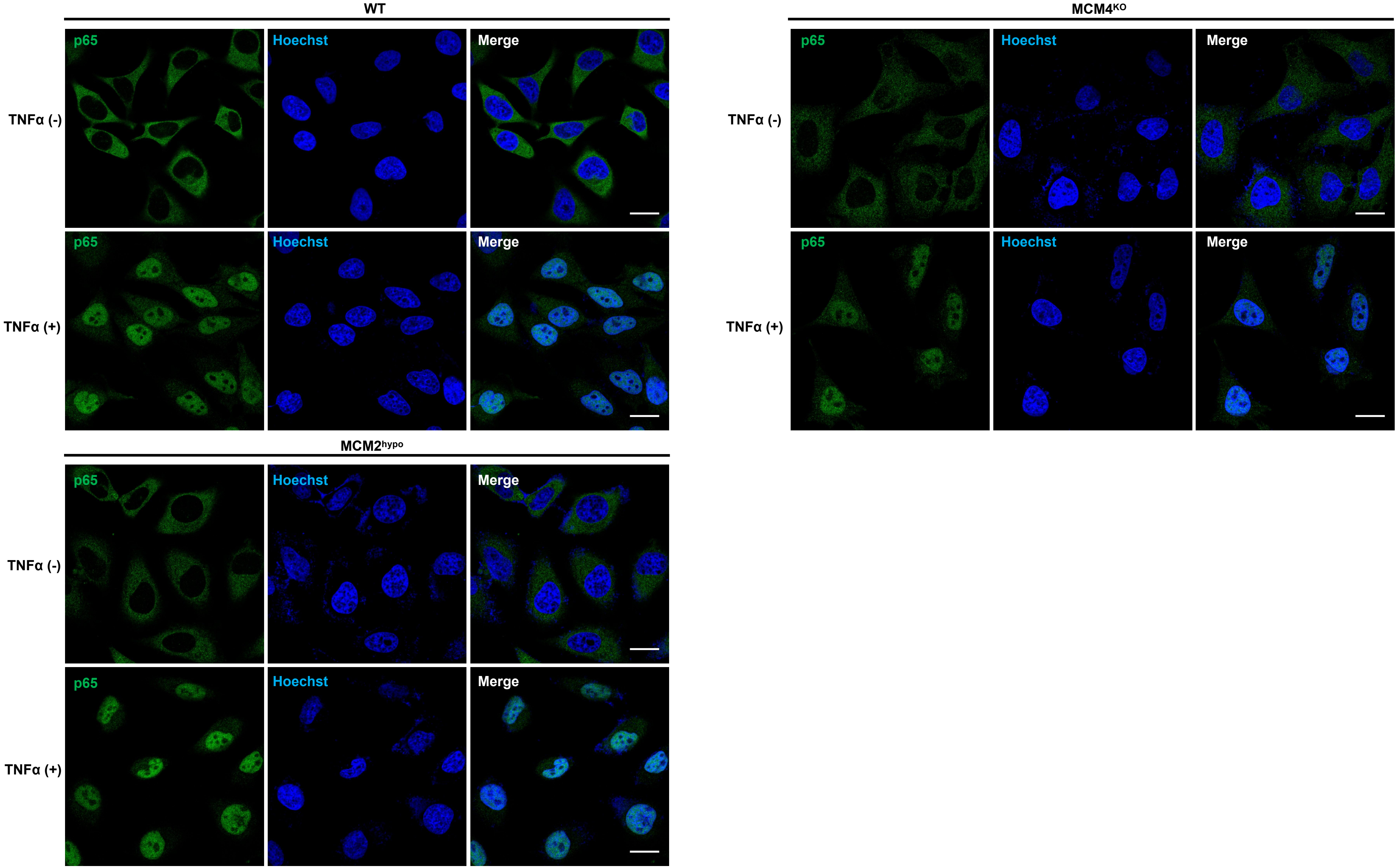

Supplementary Fig. 10 Original images of western blotting shown in Fig. 4D

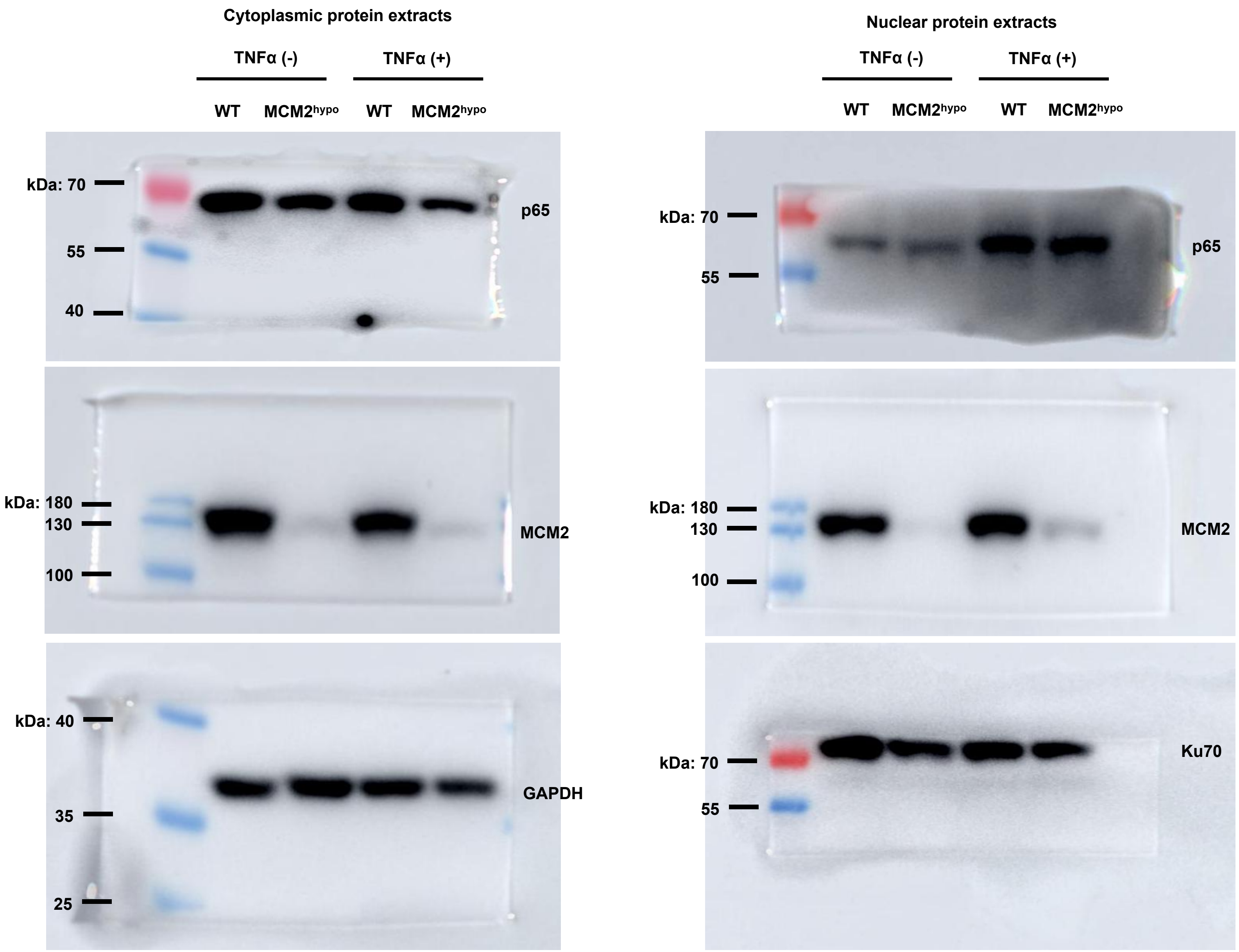

Marker： Thermo 26616

Supplementary Fig. 11 Original images of western blotting shown in Fig. 4E

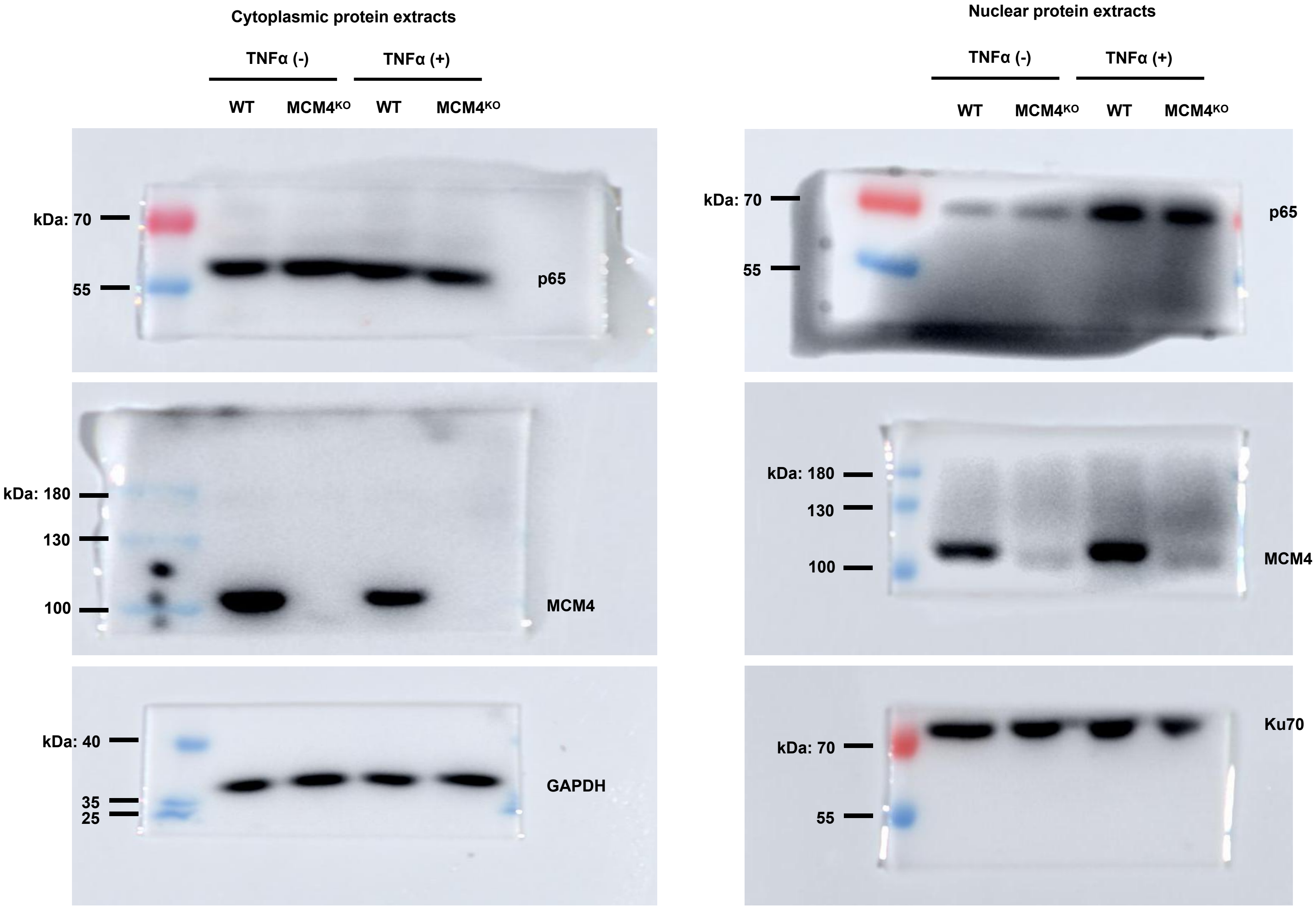

Marker：Thermo 26616

Supplementary Fig. 12 Original images of western blotting shown in Fig. 4G

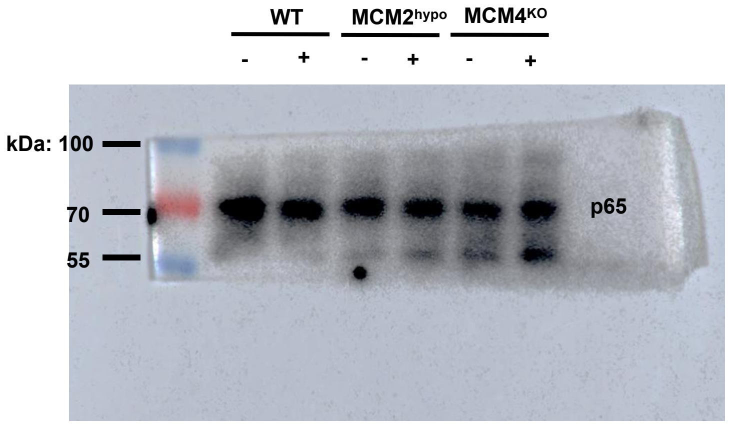

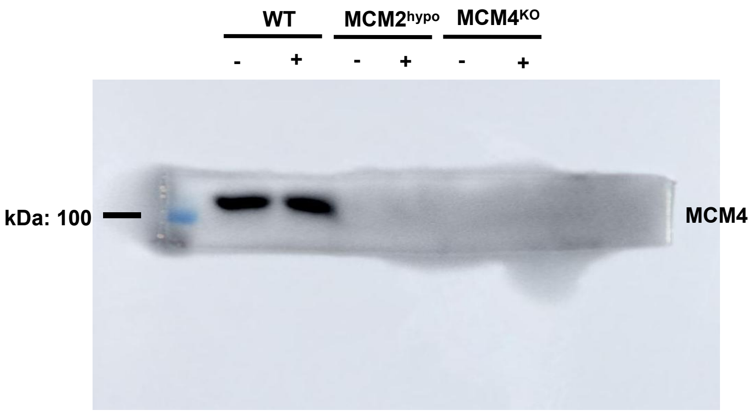

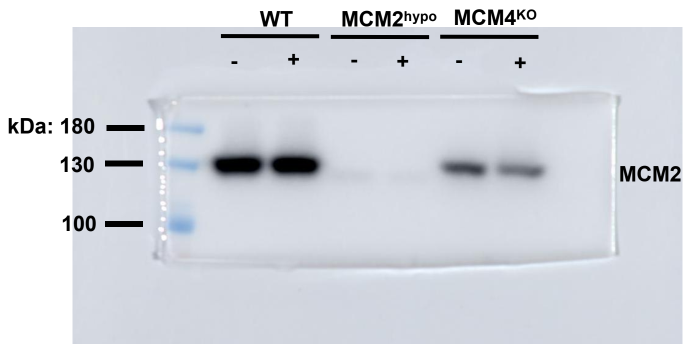

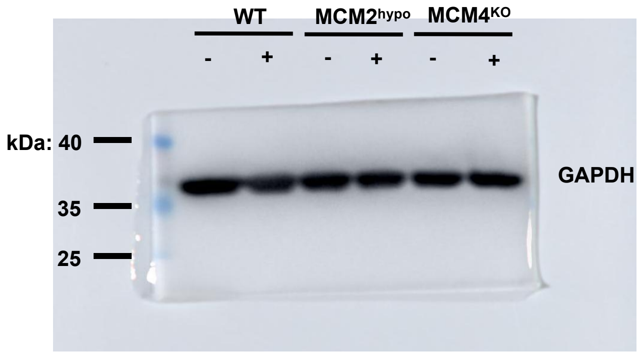

Marker：Thermo 26616

Supplementary Fig. 13 Original images of western blotting shown in Fig. 5B

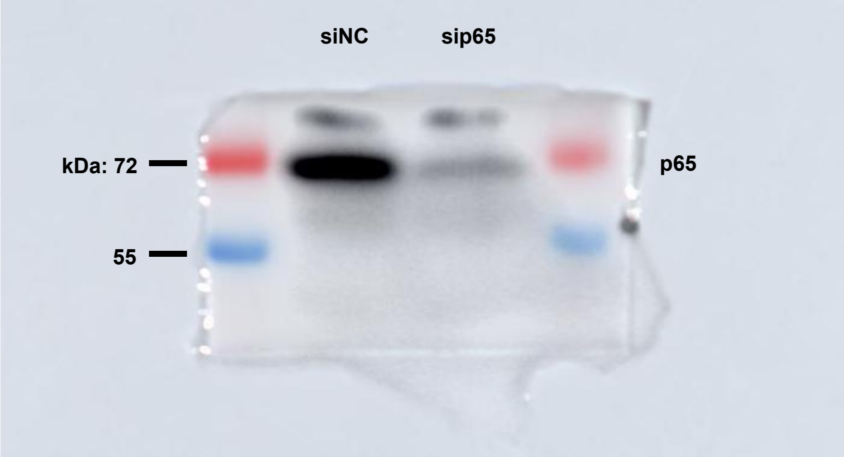

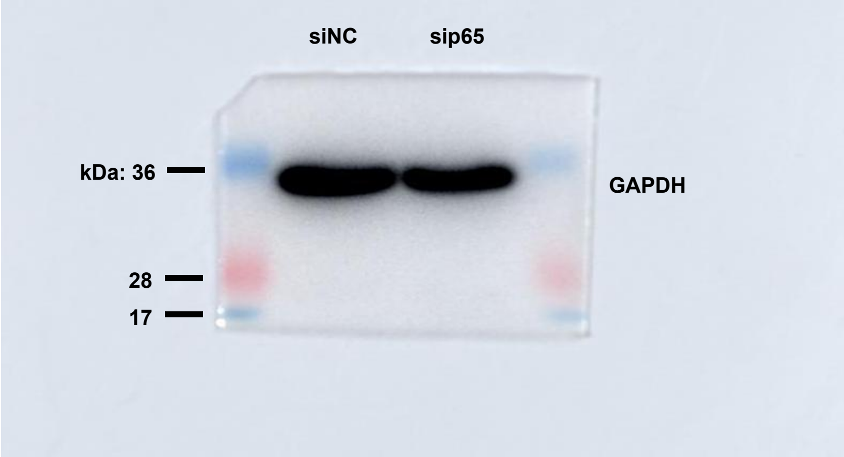

Marker: Thermo 26619

Supplementary Fig. 14 Original images of western blotting shown in Supplementary. Figure S1A

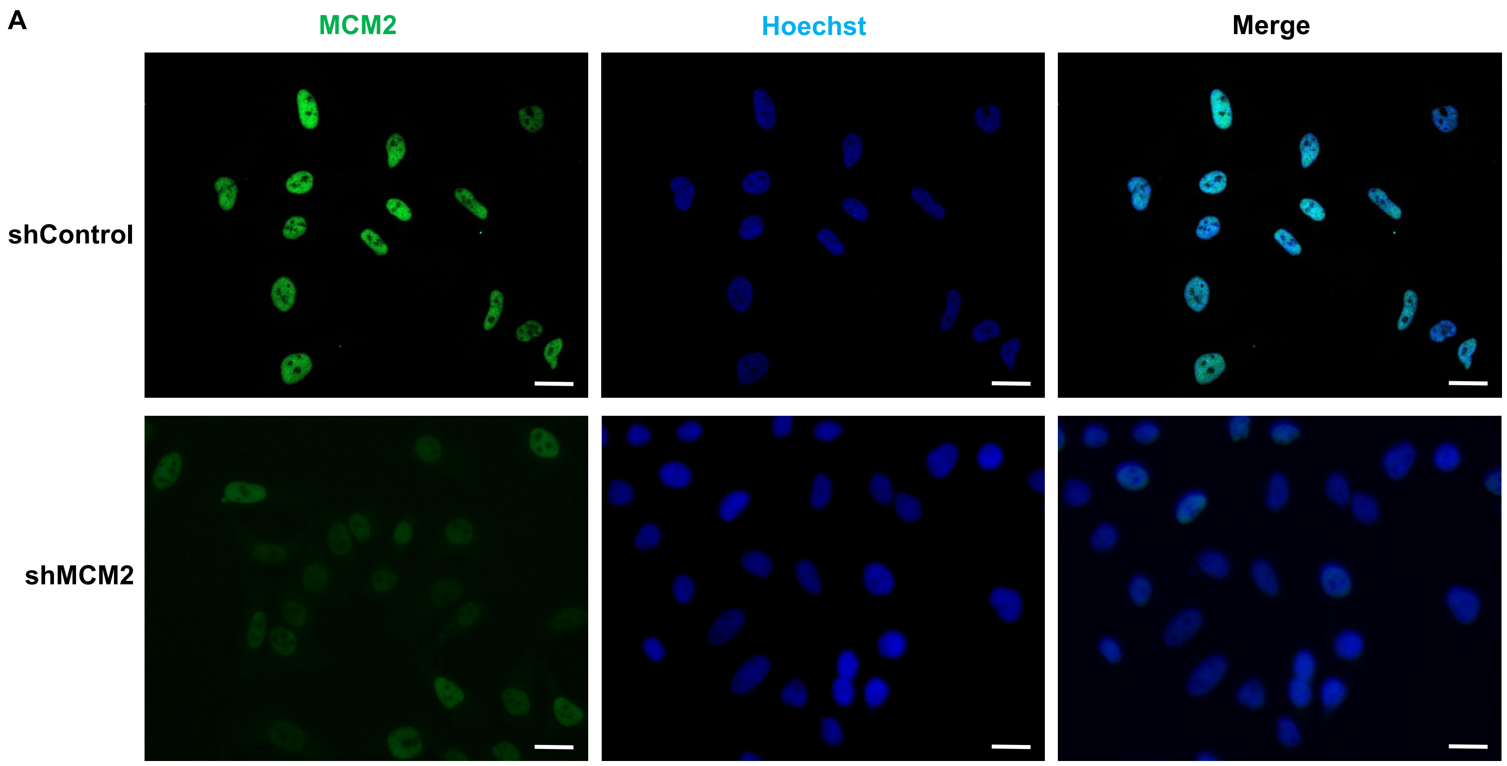

Supplementary Fig. 15 Original images of western blotting shown in Supplementary. Figure S1B

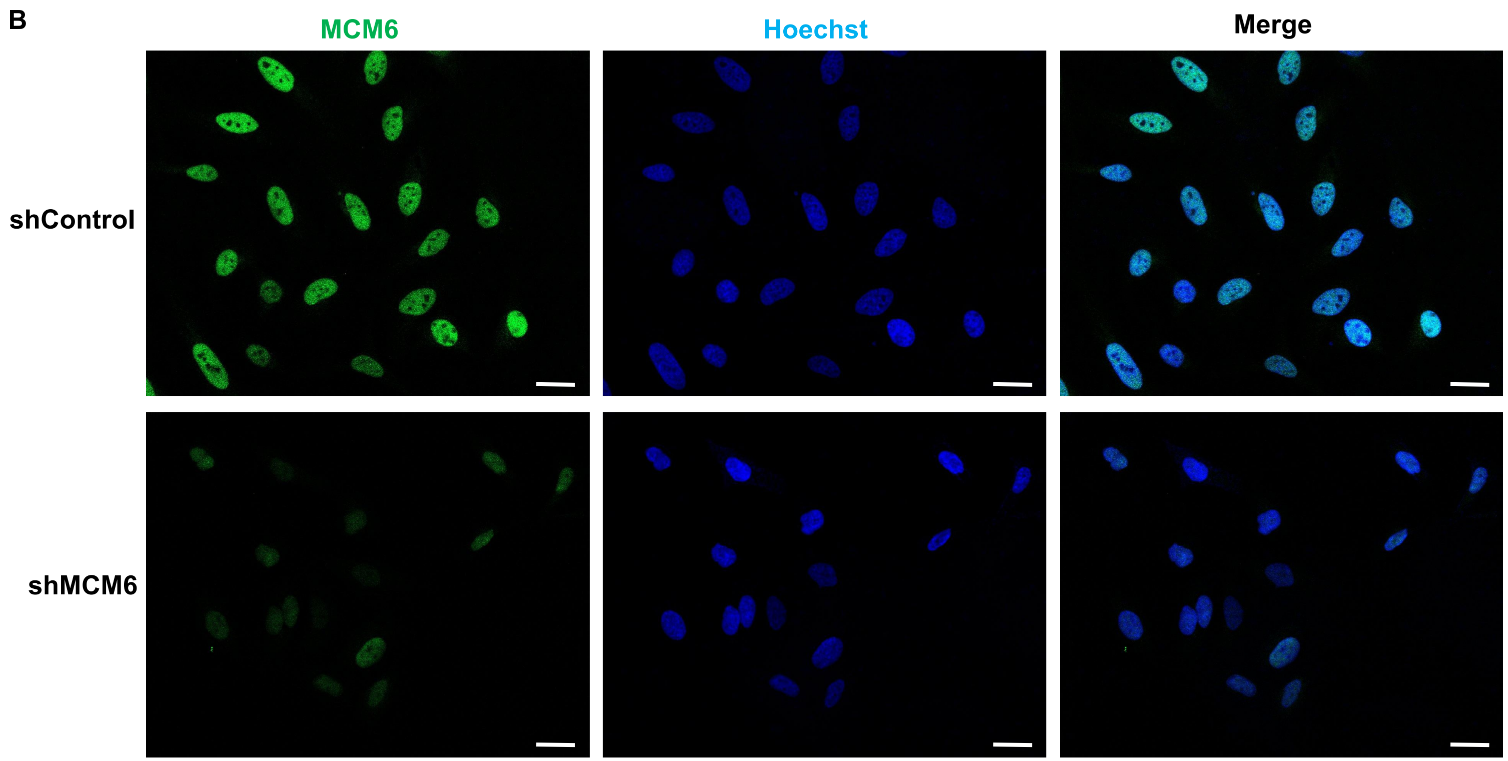

Supplementary Fig. 16 Original images of western blotting shown in Supplementary. Figure S4A

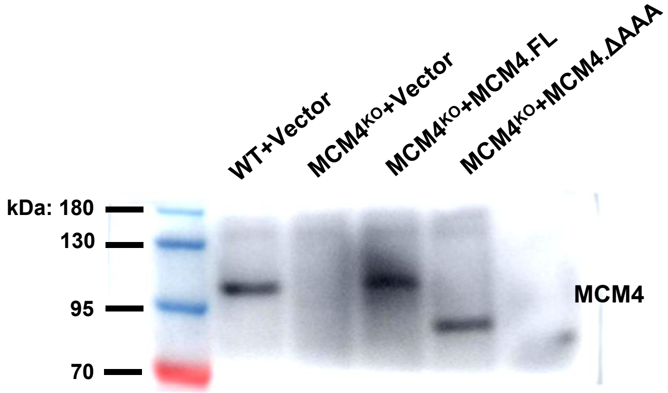

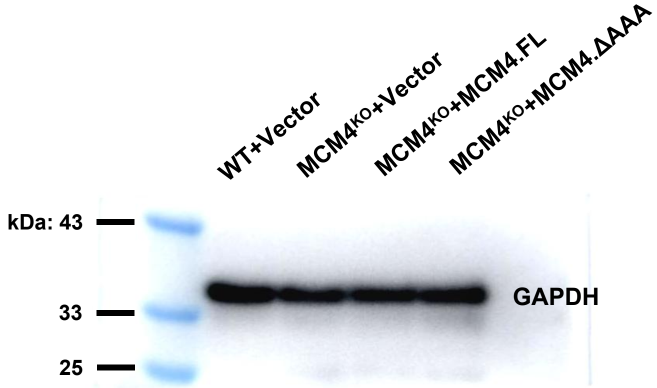

Marker：Biosharp BL712A

Supplementary Fig. 17 Original images of western blotting shown in Supplementary. Figure S5B

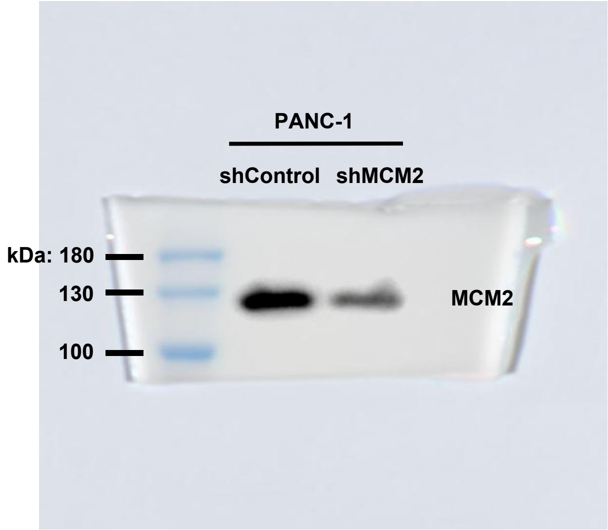

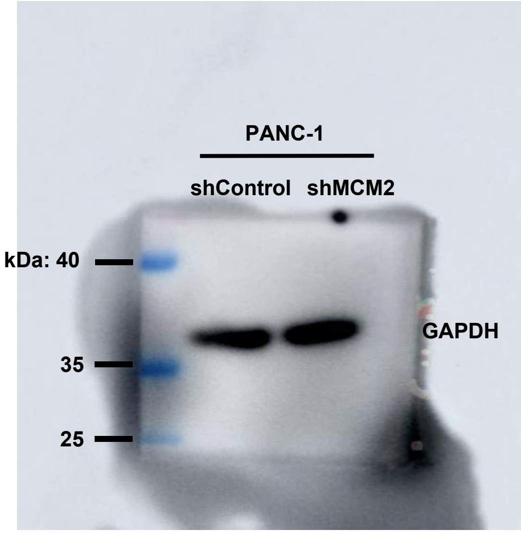

Marker: Thermo 26616

Supplementary Fig. 18 Original images of western blotting shown in Supplementary. Figure S7A

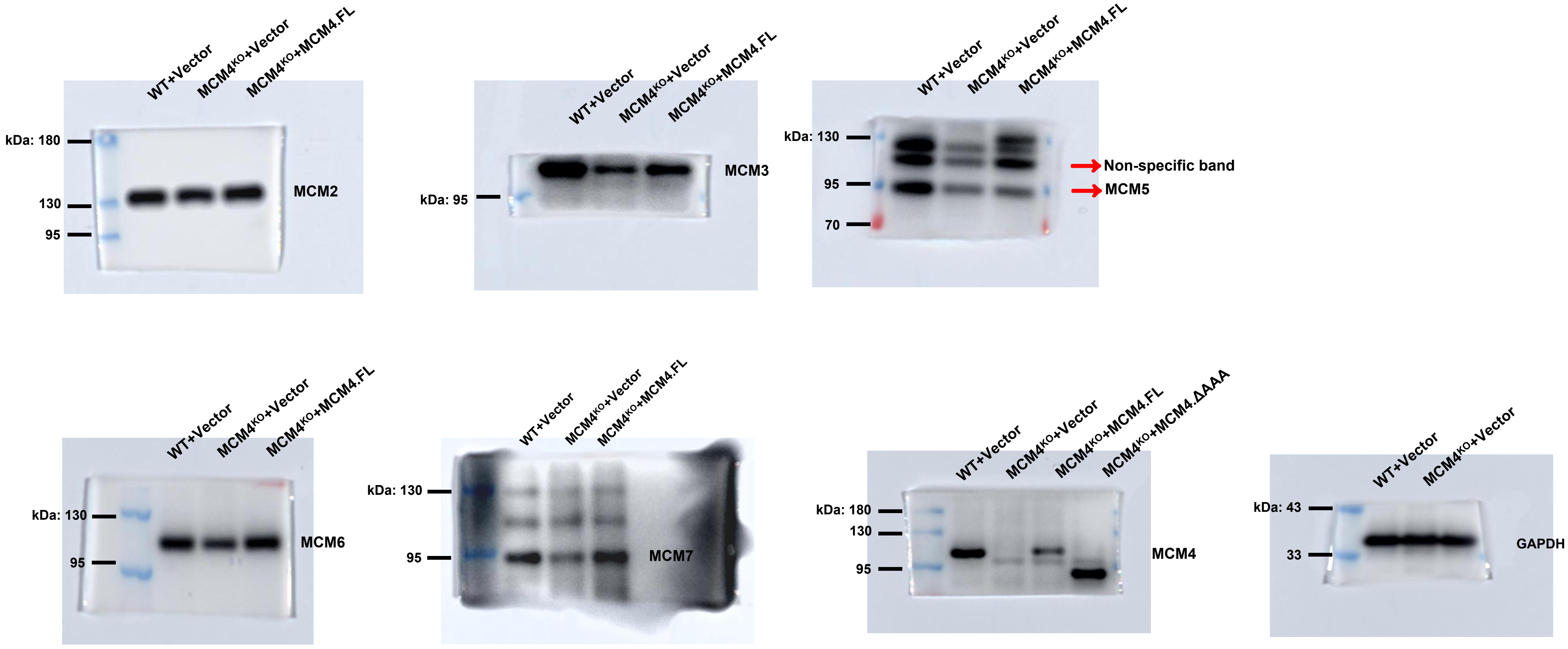

Marker: Biosharp BL712A
